# Integrated machine learning for cause-of-death classification and postmortem interval prediction: Liver and kidney metabolomics from seawater-immersed rat cadavers

**DOI:** 10.1371/journal.pone.0353958

**Published:** 2026-07-23

**Authors:** Jianghuan Lu, Yuzhao Xu, Siqi Chen, Zhiao Duan, Yixin Ma, Xiaoshi Qin, Yunchao Zhou, Shan Ha, Jianhua Chen, Jianqiang Deng

**Affiliations:** 1 Key Laboratory of Tropical Translational Medicine of Ministry of Education, School of Basic Medical Sciences, Hainan Academy of Medical Sciences, Hainan Medical University, Haikou, China; 2 Department of Forensic Medicine & Hainan Provincial Tropical Forensic Engineering Research Center & Hainan Provincial Tropical Forensic Academician Workstation, School of Basic Medical Sciences, Hainan Medical University, Haikou, China; Universiti Teknologi Malaysia - Main Campus Skudai: Universiti Teknologi Malaysia, MALAYSIA

## Abstract

**Purpose:**

To assess whether liver and kidney metabolomics combined with machine learning can distinguish seawater drowning from postmortem submersion after CO_2_ euthanasia and estimate postmortem interval (PMI) under controlled conditions.

**Methods:**

Sixty male Sprague–Dawley rats were assigned to a seawater-drowning group or a postmortem-submersion group after CO_2_ euthanasia (30 per group). Liver and kidney tissues were collected at 0, 12, 24, 36, 48, and 72 h after death and analyzed by untargeted LC–MS/MS. Principal component analysis (PCA) and cross-validated orthogonal partial least squares discriminant analysis (OPLS-DA) were used to characterize global metabolic variation. Random Forest (RF), Support Vector Machine (SVM), Multi-Layer Perceptron (MLP), and Gradient Boosting Decision Tree (GBDT) were evaluated using repeated 10-fold cross-validation (five repeats; 50 folds in total). PMI-specific XGBoost models used 20 metabolites selected within each training fold by absolute Spearman correlation and were assessed by repeated 10-fold and leave-one-time-point-out cross-validation. SHAP analysis summarized feature contributions.

**Results:**

PCA showed that dominant metabolic variation was mainly associated with PMI, with substantial overlap between the two groups in the PC1–PC2 space. Cross-validated OPLS-DA identified group-associated structure in liver and kidney, with Q^2^(cum) values of 0.847 and 0.723, respectively. Mean classification AUCs ranged from 0.973 to 0.996 in liver and from 0.931 to 1.000 in kidney. Under repeated 10-fold cross-validation, the liver and kidney PMI models achieved MAEs of 4.78 and 4.05 h and R^2^ values of 0.823 and 0.892, respectively. Under leave-one-time-point-out cross-validation, MAEs increased to 13.52 and 13.64 h, while R^2^ values were 0.587 and 0.592. SHAP analysis showed partly different metabolite contribution patterns between the two organs.

**Conclusion:**

In this controlled rat model, liver and kidney metabolomic profiles combined with machine learning supported discrimination between seawater drowning and postmortem submersion after CO_2_ euthanasia and captured PMI-related metabolic changes. The classification models, PMI-specific XGBoost regression models, and SHAP analyses characterized organ-specific metabolite patterns associated with discrimination between the two modeled conditions and PMI prediction.

## Introduction

In forensic practice, determining whether drowning was the cause of death is a central question when examining bodies recovered from water [1]. The diagnosis of drowning commonly relies on postmortem findings and diatom testing [1,2]. However, these approaches have limited specificity, and diatom testing is vulnerable to contamination and both false-positive and false-negative results, particularly in seawater cases [2,3]. More objective methods are needed to distinguish seawater drowning from postmortem submersion and to support postmortem interval (PMI) estimation. Although both conditions involve seawater exposure, seawater drowning may involve aspiration, impaired gas exchange, and hypoxia, whereas postmortem submersion occurs without antemortem aspiration and mainly reflects postmortem and environmental changes [4]. This contrast provides the basis for testing whether organ metabolomic profiles can distinguish the two conditions and capture PMI-related variation.

Postmortem metabolite levels change over time, providing a basis for metabolomics-based PMI estimation [5,6]. PMI is also a major potential confounder in postmortem metabolomic comparisons [7]. Recent studies have investigated PMI estimation using metabolite changes in serum, aqueous humor, vitreous humor, and other fluids [8,9]; human femoral blood and bone have also been examined using postmortem metabolomic or multi-omic approaches [10,11]. Metabolomic studies of submerged cadavers have examined blood and skeletal muscle, whereas metabolomic studies of visceral organs in the context of seawater drowning remain limited [12,13]. Liver and kidneys were selected as metabolically active solid organs. Both organs have been included in multi-organ PMI studies, and time-dependent molecular changes have been documented in postmortem liver and kidney tissues [14,15]. However, their proximity to the gastrointestinal tract may increase their exposure to postmortem bacterial spread, and intestine-derived bacteria can contribute to microbial communities in submerged liver tissue [16,17]. By contrast, the brain is more anatomically isolated from the gastrointestinal tract and may provide a useful comparator in future multi-organ studies, although it was not sampled in the present experiment. We therefore established seawater-drowning and postmortem-submersion groups under the same seawater exposure conditions to compare liver and kidney metabolomic profiles between the two conditions and across PMI. The analysis was limited to liver and kidney tissues and did not include lung tissue or diatom testing. The observed metabolite differences were therefore interpreted as features distinguishing the two experimental conditions rather than as biomarkers specific to seawater drowning.

In this study, we characterized liver and kidney metabolomic profiles in rats subjected either to seawater drowning or to CO_2_ euthanasia followed by postmortem submersion under controlled conditions. Untargeted LC-MS/MS, multivariate analysis and machine-learning models were used to evaluate discrimination between the two groups and PMI prediction separately in liver and kidney. Metabolites associated with classification or PMI prediction were treated as model-associated candidates rather than validated forensic biomarkers and require independent confirmation before forensic use.

## Materials and methods

### Animal model preparation

Sixty male Sprague-Dawley (SD) rats (6–7 weeks old, weighing 220–250 g) were randomly assigned to a seawater-drowning group (D group) and a postmortem submersion group (PS group), with 30 rats in each group. A prespecified termination criterion was applied: observation was discontinued if decomposition-related disruption of the thoracic or abdominal skin exposed the body cavity, because the integrity of liver and kidney could no longer be assured. Pilot observations indicated that this condition generally occurred after 72 h postmortem. Sampling was therefore performed at 0, 12, 24, 36, 48, and 72 h, with five animals at each time point in each group. The seawater used in the experiment was collected from coastal waters off Hainan Island. To minimize changes before use, the seawater was transported and stored at 4°C in the dark and used within 12 h of collection. All experiments were conducted in a controlled climatic chamber maintained at 25°C and 80% relative humidity (RH), conditions selected to approximate the environmental conditions of Hainan Island (annual temperature 23.1–27.0°C; humidity 80–85% RH).

Seawater drowning group (D Group): No anaesthetic or sedative was administered before the drowning procedure. Each rat was placed in sterile mesh bags, submerged in seawater for 1 min, and then removed from the water for 30 s. This cycle was repeated until respiration ceased and death was confirmed. The carcasses were then transferred to a transparent aquarium filled with seawater and maintained in the climatic chamber until sampling.

Postmortem submersion group (PS Group): Thirty rats were euthanized by CO_2_ exposure in sealed plastic chambers. CO_2_ was introduced from a cylinder for approximately 5 min until apnea, pupillary dilation, and cessation of heartbeat were observed. After a further 2-min observation period to confirm death, the carcasses were submerged in seawater and maintained in the climatic chamber until sampling. The procedure was adapted from previously reported rat models of postmortem submersion and used the same seawater and climatic conditions as the drowning group [12,13].

The study was approved by the Animal Welfare and Ethics Review Board of Hainan Medical University (Approval No.: HYLL-2021–346).

### Sample collection

At each of the six fixed time points (0 h, 12 h, 24 h, 36 h, 48 h, and 72 h after death), five rat corpses were randomly selected from each experimental group (D group and PS group), resulting in a total of ten animals per time point. All dissections were performed in a laminar-flow cabinet to minimize external contamination. Surgical instruments were sterilized by autoclaving at 121 °C for 30 minutes before use. Separate sets of sterilized instruments were used for skin incision, liver collection, and kidney collection. Cryogenic storage tubes were autoclaved and depyrogenated before use. The harvested livers and kidneys were immediately flash-frozen in liquid nitrogen and subsequently stored at −80 °C for further analyses.

### Sample pretreatment

Sample preparation and LC–MS/MS analysis were performed at Shanghai Biotree Biomedical Technology Co., Ltd. Under chilled conditions, liver and kidney samples were weighed into microcentrifuge tubes, containing homogenization beads and 500 μL of an isotope-labelled internal-standard extraction solution (methanol/acetonitrile/water = 2:2:1, v/v/v). Samples were homogenized at 35 Hz for 4 min and sonicated in an ice-water bath for 5 min; the sonication procedure was performed twice. Samples were then incubated at –40 °C for 1 h and centrifuged at 13,800 × g and 4 ℃ for 15 minutes. The resulting supernatant was transferred into autosampler vials for LC-MS/MS analysis. Additionally, a pooled quality control (QC) sample was prepared by mixing equal aliquots of the supernatants from all samples and was repeatedly analyzed throughout the LC–MS/MS run to monitor analytical stability [18,19].

### LC–MS/MS analysis and metabolite annotation

A Vanquish ultra-high performance liquid chromatography system was coupled to an Orbitrap Exploris 120 mass spectrometer and operated using Xcalibur version 4.4 (Thermo Fisher Scientific, USA). Chromatographic separation was performed using a Waters ACQUITY UPLC BEH Amide column (2.1 mm × 50 mm, 1.7 µm). The mobile phase consisted of solvent A (water with 25 mmol/L ammonium acetate and 25 mmol/L ammonia) and solvent B (acetonitrile). The autosampler temperature was maintained at 4 °C, and the injection volume was 2 µL. Mass spectrometry acquisition parameters were as follows: sheath gas flow rate, 50 Arb; auxiliary gas flow rate, 15 Arb; capillary temperature, 320 °C; spray voltage, + 3.8 kV (positive mode) or −3.4 kV (negative mode); full MS resolution, 60,000; MS/MS resolution, 15,000; and stepped normalized collision energies of 20, 30, and 40. Raw data files were converted to mzXML format using ProteoWizard and subsequently processed for metabolite annotation using an in-house R package provided by Shanghai Biotree Biomedical Technology Co., Ltd., together with the BiotreeDB database (version 3.0) [20]. Annotation confidence was categorized according to the MSI-based scheme implemented in the BiotreeDB/MetDNA2 workflow [20,21]. Level 1 annotations were supported by matches of MS1, retention time, and MS/MS spectra to in-house chemical standards. Level 2 annotations were based on MS1 and MS/MS matches to public spectral libraries without retention-time confirmation. Level 3 annotations were based on MS1, predicted retention time, and surrogate MS/MS spectra and were further subdivided by the workflow into Level 3.1 for putatively annotated known database compounds and Level 3.2 for putatively annotated predicted structures. The MS2 name field was used as the metabolite label in subsequent analyses, whereas the corresponding annotation level was retained separately to indicate annotation confidence.

### Data processing and statistical analysis

Peak tables were processed using established untargeted-metabolomics quality-control and preprocessing principles [21,22]. (1) Outlier filtering: Features with an RSD greater than 30% across pooled QC samples were excluded. (2) Missing value filtering: Features more than 50% missing values in any group or in the complete dataset were removed. (3) Missing value imputation: Remaining missing values were replaced with one-half of the minimum positive value recorded for the corresponding feature. (4) Data normalization: Feature areas were normalized to the total ion current (TIC) of each sample.

Principal component analysis (PCA) and orthogonal partial least squares discriminant analysis (OPLS-DA) were performed using SIMCA version 16.0.2 (Sartorius Stedim Data Analytics AB, Umeå, Sweden). SIMCA was used so that PCA, OPLS-DA, cross-validation, CV-ANOVA, and response-permutation testing could be conducted within the same chemometric workflow. Both analyses used the same log10-transformed and unit-variance-scaled metabolite matrix. The R-based workflow described above was used for metabolite annotation and initial data processing, whereas Python was used for subsequent statistical analyses, machine-learning modeling, and figure generation. Before analysis, metabolite data were log10-transformed and unit variance scaled. Figures were generated in Python based on the corresponding SIMCA analysis results.

PCA was first applied to characterize the overall variation in the metabolic profiles. To determine whether group-related variation was present across principal components beyond PC1 and PC2, the scores of PC1–PC10 were analyzed separately for liver and kidney. For each principal component, a two-factor linear model included group, PMI, and the group × PMI interaction, with both group and PMI treated as categorical factors. Type III analysis of variance with sum contrasts was used to test the two main effects and their interaction. P values were adjusted across PC1–PC10 using the Benjamini–Hochberg procedure separately for each organ and each effect, and partial eta squared was calculated as the effect-size measure. These analyses were conducted in Python 3.10.20 using statsmodels version 0.14.6. Type III tests were implemented with sum contrasts.

OPLS-DA was then used to examine D/PS-associated structural variation in the liver and kidney metabolite profiles. Model performance was evaluated using five-fold cross-validation, with R^2^X(cum), R^2^Y(cum), and Q^2^(cum) used to describe the explained X variance, model fit, and cross-validated predictive ability, respectively. CV-ANOVA was performed using the cross-validated residuals, and the distribution expected under random class assignment was examined using 200-response permutation tests conducted under the same validation settings [23]. The R^2^ and Q^2^ regression intercepts were recorded, and the original model values were compared with those obtained from the permuted-label models. Cross-validated predictive and orthogonal scores were used for the OPLS-DA score plots.

### Differential metabolite screening and KEGG pathway mapping

The D0h and PS0h groups were compared separately in liver and kidney using the metabolite-level fold-change, P-value, and FDR results generated during statistical processing. These tables contained metabolite annotations, log₂FC values, P values, FDR-adjusted P values, KEGG COMPOUND IDs when available, and annotation levels. Annotation levels were retained from the metabolite annotation output to indicate the relative confidence of metabolite identification; they were used for reporting purposes and were not treated as independent experimental validation. Metabolites were defined as differential metabolites only when they met both FDR < 0.05 and |log₂FC| > 1. Log₂FC was calculated as D0h relative to PS0h; positive values indicated higher metabolite abundance in D0h, whereas negative values indicated lower abundance in D0h. Volcano plots were generated using the log₂FC and FDR values, and metabolites that did not meet both criteria were shown as non-significant.

KEGG pathway mapping was performed to identify the pathways containing the differential metabolites. KEGG COMPOUND IDs of the differential metabolites were matched to the corresponding KEGG pathway mapping records separately for liver and kidney. The mapping output included the mapped metabolite names, KEGG compound IDs, annotation levels, and the direction of change for each metabolite in the D0h versus PS0h comparison. This was a descriptive mapping analysis and was not used to infer pathway enrichment, activation, or inhibition. The reported directions refer to individual metabolites rather than pathway-level regulation.

### Machine learning algorithms

Cause-of-death classification was performed separately in liver and kidney by pooling the six PMI levels within each organ and distinguishing D from PS samples. Random Forest (RF), Support Vector Machine (SVM), Multi-Layer Perceptron (MLP), and Gradient Boosting Decision Tree (GBDT) models were evaluated under the same validation framework. RF and GBDT represented tree-based ensemble methods, SVM provided a margin-based classifier for the high-dimensional feature space, and MLP served as a neural-network comparator. The comparison was intended to cover distinct nonlinear model families rather than all available classifiers.

Because each rat contributed one tissue sample at a single predefined PMI, data splitting was performed at the animal level. Model performance was evaluated using repeated stratified 10-fold cross-validation with five repeats, producing 50 validation folds. Within each fold, metabolite intensities were standardized using parameters estimated from the training subset and then applied to the corresponding validation subset. Model hyperparameters were prespecified and kept fixed across folds. For the primary RF analysis, feature importance was calculated separately in each training fold. Mean importance, rank variability, and top-20 selection frequency were then summarized across the 50 folds. A PMI-adjusted sensitivity analysis was also conducted by removing PMI-specific mean shifts within the training data and applying the corresponding adjustment to the validation data before model fitting.

PMI regression pooled the D and PS samples within each organ and was performed separately for liver and kidney. XGBoost was selected to model nonlinear metabolite–PMI associations while constraining model complexity through shrinkage, subsampling, and regularization [24]. Within each training fold, Spearman correlations between metabolite intensity and PMI were calculated using the training samples only. The 20 metabolites with the largest absolute correlation coefficients were selected for model fitting and prediction of the corresponding validation samples.

XGBoost regression was implemented in Python 3.10 using xgboost version 3.2.0. The same hyperparameter settings were applied to both organs: 100 trees, a maximum tree depth of 5, a learning rate of 0.2, subsample and colsample_bytree values of 0.8, gamma of 0.1, L1 regularization of 0.05, and L2 regularization of 1.0. The L1 and L2 terms were used to place mild constraints on terminal-node weights and model complexity rather than to perform additional feature selection among the 20 input metabolites. Squared-error regression was used as the objective function. All hyperparameters were specified before model fitting and were not tuned using the present dataset; therefore, this parameter combination was not considered an optimized setting. Model performance was assessed using repeated 10-fold cross-validation with five repeats, producing 50 validation folds. In each fold, metabolite selection, model fitting, and prediction were confined to the corresponding training–validation split. TreeSHAP contribution values were also calculated for the fold-specific top-20 features in the validation samples, and mean absolute contribution values and within-fold ranks were summarized across the 50 validation folds to assess feature-contribution stability [25]. Performance was evaluated using R^2^, root mean square error (RMSE), mean squared error (MSE), mean absolute error (MAE), and mean absolute percentage error (MAPE). Samples with a PMI of 0 h were excluded from MAPE calculation. Fold-level results were reported as mean values with 95% confidence intervals.

For visualization of repeated 10-fold cross-validation results, each sample generated one out-of-fold prediction in each of the five repeats. The five predictions were averaged to obtain one mean OOF prediction per sample for the actual-versus-predicted plots. These sample-level mean predictions were used only for visualization, whereas repeated-cross-validation performance was summarized from the 50 fold-level estimates.

To evaluate the influence of shared PMI time points between training and validation sets, leave-one-time-point-out cross-validation was also performed. In each iteration, all samples from one PMI time point were withheld, and the remaining time points were used for feature selection and model training. The trained model was then applied to the withheld time point. This procedure was repeated for all six PMI levels, and the pooled predictions from the six held-out time points were used to calculate overall performance. Prediction bias was summarized by PMI time point using prediction error, defined as predicted PMI minus actual PMI.

For descriptive model interpretation, metabolites were ranked by the absolute Spearman correlation with PMI in the complete organ-specific dataset, and the global top 20 were used to refit one final XGBoost model per organ. These full-data models were not used to estimate predictive performance. TreeSHAP contribution values were calculated using the native XGBoost implementation, and the final models were summarized using mean absolute TreeSHAP values, beeswarm plots, dependence plots, and gain-based feature importance. Spearman P values for the full-data metabolite–PMI correlations were adjusted using the Benjamini–Hochberg procedure. These correlations and final-refit interpretation outputs were treated as descriptive analyses rather than independent validation results.

The classification and PMI regression analyses were conducted in Python 3.10 using scikit-learn version 1.7.1, SciPy version 1.15.3, and XGBoost version 3.2.0. TreeSHAP contribution values were calculated using the native XGBoost implementation. A separate requirements.txt file containing pinned dependencies for rerunning the deposited scripts is provided in the Zenodo repository.

## Results

### Metabolite annotation profile in liver and kidney

LC–MS/MS analysis yielded 2,527 and 2,902 metabolite annotation entries in liver and kidney tissues, respectively. In liver, 529 annotations were assigned to Level 1 (20.9%), 1,845 to Level 2 (73.0%), and 153 to Level 3 (6.1%), including 97 Level 3.1 and 56 Level 3.2 annotations. In kidney, 559 annotations were assigned to Level 1 (19.3%), 2,155 to Level 2 (74.3%), and 188 to Level 3 (6.5%), including 109 Level 3.1 and 79 Level 3.2 annotations (S11 Table).

**Fig 1:**
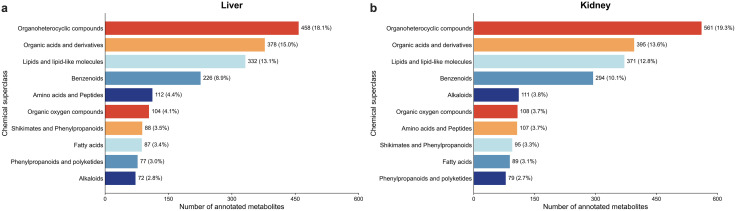
Distribution of the 10 most frequently represented chemical superclasses among metabolite annotation entries in liver and kidney tissues. (a) Liver; (b) kidney. Bars indicate the number of annotation entries assigned to each chemical superclass, and labels show the corresponding count and percentage. The summary includes Level 1, Level 2, Level 3.1, and Level 3.2 annotations. Percentages were calculated from annotation counts and do not represent relative metabolite abundance.

The three most frequent chemical superclasses were organic heterocyclic compounds (18.1% in liver and 19.3% in kidney), organic acids and derivatives (15.0% and 13.6%, respectively), and lipids and lipid-like molecules (13.1% and 12.8%, respectively). The corresponding differences between organs were 1.2, 1.4, and 0.3 percentage points, and the rank order of the three superclasses was identical in liver and kidney (Fig 1).

### Global metabolic variation and D/PS group structure

**Fig 2:**
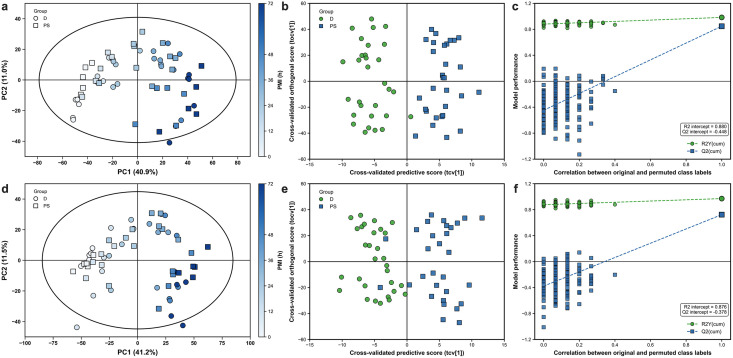
PCA and cross-validated OPLS-DA analyses of liver and kidney metabolic profiles. (a–c) Liver; (d–f) kidney. (a, d) PCA score plots, with marker shapes indicating the drowning (D) and postmortem submersion (PS) groups and the light-to-dark colour gradient indicating increasing postmortem interval from 0 to 72 h. Ellipses represent 95% confidence regions. (b, e) Five-fold cross-validated OPLS-DA score plots. Green circles indicate D samples and blue squares indicate PS samples; tcv [1] and tocv [1] denote the cross-validated predictive and orthogonal scores, respectively. (c, f) Results of 200-response permutation tests. Green circles and blue squares represent R^2^Y(cum) and Q^2^(cum), respectively, and dashed lines indicate the corresponding regression lines. The points at a correlation of 1 represent the original models.

In liver, PC1 and PC2 explained 40.9% and 11.0% of the total variance, respectively, whereas the corresponding values in kidney were 41.2% and 11.5%. The first ten principal components cumulatively explained 77.8% of the variance in liver and 77.0% in kidney (S1 Table). In both organs, the PC1–PC2 plots showed ordering along the PMI gradient but substantial overlap between D and PS samples (Fig 2a and 2d).

Analysis of the exported PC scores showed that PMI effects were significant for PC1 and PC2 in both organs, whereas neither component showed an FDR-significant D/PS group effect. After accounting for PMI, significant D/PS group effects were detected in liver PC4, PC6, and PC7 and in kidney PC3, PC5, PC6, and PC7. FDR-significant group × PMI interactions were observed only in kidney PC5, PC7, and PC9; no interaction remained significant in the liver after FDR correction (S1 Table). QC samples formed tight clusters in both datasets (S1 Fig. a and b).

Five-fold cross-validated OPLS-DA score plots showed partial D/PS separation in both organs, with greater overlap in kidney (Fig 2b and 2e). Q^2^(cum) was 0.847 for liver and 0.723 for kidney. CV-ANOVA was significant for both models (liver, p = 1.23 × 10 ⁻ ¹⁶; kidney, p = 1.41 × 10 ⁻ ¹⁰). In the 200-response permutation tests, the original Q^2^ values exceeded those obtained after label permutation, and the Q^2^ intercepts were negative in liver (−0.448) and kidney (−0.378) (Fig 2c and 2f). The R^2^ intercepts remained high in both liver (0.880) and kidney (0.876), showing that permuted-label models could retain substantial apparent fit despite the negative Q^2^ intercepts. Full model and validation parameters are provided in S2 Table.

### Differential metabolic profiles between drowning and postmortem-submersion groups

**Fig 3:**
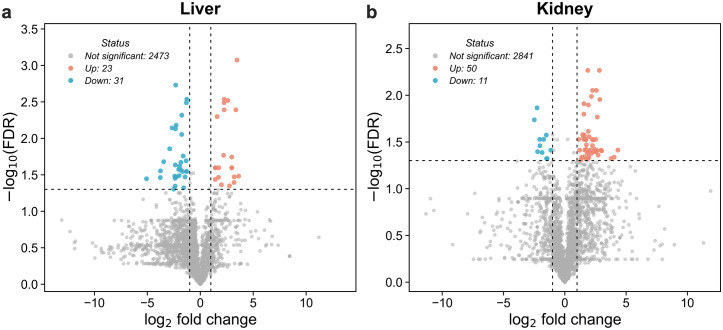
Differential metabolite profiles in D0h versus PS0h. Volcano plots showing differential metabolites in liver (a) and kidney (b) tissues between the drowning group at 0 h (D0h) and the postmortem-submersion control group at 0 h (PS0h). The x-axis represents log₂ fold change for D0h versus PS0h, and the y-axis represents −log₁₀(FDR). Differential metabolites were defined as those meeting both FDR < 0.05 and |log₂FC| > 1. Upregulated metabolites in D0h versus PS0h are shown in orange, downregulated metabolites in D0h versus PS0h are shown in blue, and metabolites that did not meet both criteria are shown in grey.

At 0 h, 54 annotated liver metabolites met the predefined criteria of FDR < 0.05 and |log₂FC| > 1; 23 showed higher abundance and 31 showed lower abundance in D0h than in PS0h (Fig 3a). In kidney, 61 annotated metabolites met the same criteria; 50 showed higher abundance and 11 showed lower abundance in D0h than in PS0h (Fig 3b).

KEGG COMPOUND identifiers were available for 12 of the 54 liver differential metabolites and 9 of the 61 kidney differential metabolites. Nine liver metabolites and five kidney metabolites mapped to at least one KEGG pathway, yielding 16 liver and 24 kidney pathway entries, respectively (S3 Table). Because several metabolites mapped to multiple entries, the number of pathway entries does not represent the same number of independent pathway changes. These directions describe individual metabolite differences and do not indicate pathway-level activation or inhibition.

### Classification models for cause-of-death discrimination

**Fig 4:**
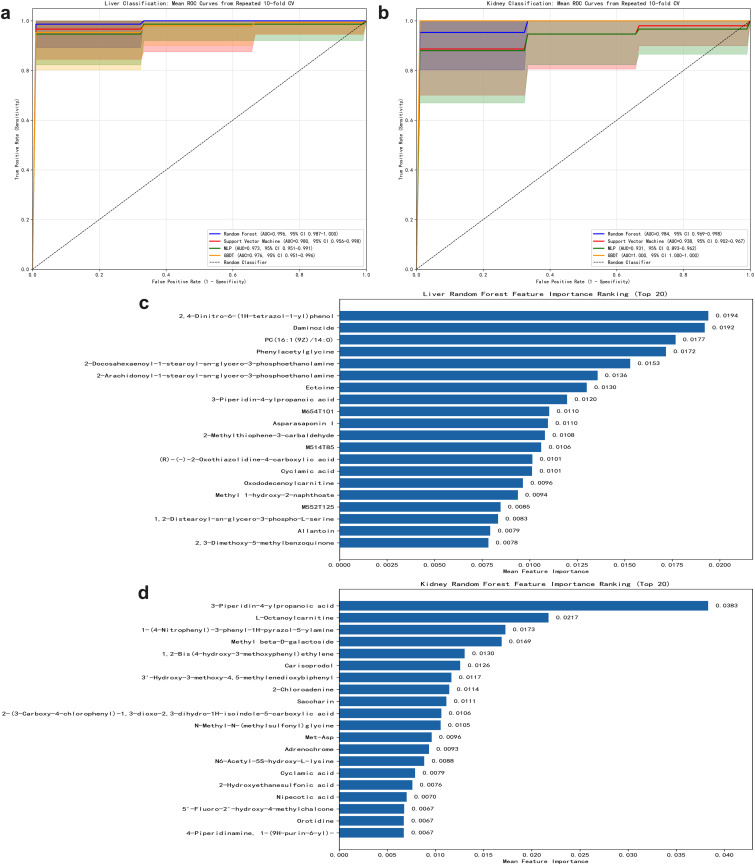
Performance of classification models and RF feature importance ranking for cause-of-death discrimination. (a, b) Mean receiver operating characteristic (ROC) curves with 95% confidence intervals (CIs) for four classification models—Random Forest (RF), Support Vector Machine (SVM), Multi-Layer Perceptron (MLP), and Gradient Boosting Decision Tree (GBDT)—developed using liver (a) and kidney (b) metabolomic data under repeated 10-fold cross-validation (five repeats; 50 folds in total). (c, d) Top 20 metabolites ranked by mean RF feature importance across the 50 folds for liver (c) and kidney (d), representing the main RF-ranked features associated with cause-of-death discrimination.

The mean receiver operating characteristic (ROC) curves with 95% confidence intervals (CIs) are presented in Fig 4a (liver) and 4b (kidney). Across the 50 validation folds, RF had the highest mean AUC in liver at 0.996 (95% CI: 0.987–1.000), followed by SVM (AUC = 0.980, 95% CI: 0.956–0.998), GBDT (AUC = 0.976, 95% CI: 0.951–0.996), and MLP (AUC = 0.973, 95% CI: 0.951–0.991). In kidney, GBDT had the highest mean AUC at 1.000 (95% CI: 1.000–1.000), followed by RF (AUC = 0.984, 95% CI: 0.969–0.998), SVM (AUC = 0.938, 95% CI: 0.902–0.967), and MLP (AUC = 0.931, 95% CI: 0.893–0.962). Accuracy, precision, recall, F1 score, and AUC across the 50 validation folds are reported in S4 Table. Fig 4c and 4d show the top 20 metabolites ranked by mean RF feature importance across the 50 folds. After removal of PMI-specific mean shift, mean AUCs ranged from 0.980 to 0.987 in liver and from 0.936 to 0.993 in kidney (S2 Fig. a and b; S5 Table). The corresponding ranges in the primary analysis were 0.973–0.996 and 0.931–1.000, respectively. RF top-20 selection frequencies and rank variability across the 50 folds are reported in S2 Fig. c,d and S6 Table. Confusion matrices for the liver and kidney classifiers are shown in S3 and S4 Figs., respectively.

### PMI regression performance and feature contributions

Under repeated 10-fold cross-validation with fold-specific Spearman top-20 selection, the liver model achieved an MAE of 4.78 h (95% CI: 4.01–5.54), RMSE of 6.66 h (95% CI: 5.66–7.65), MAPE of 16.3% (95% CI: 13.55–19.05), and R^2^ of 0.823 (95% CI: 0.732–0.914). The kidney model achieved an MAE of 4.05 h (95% CI: 3.27–4.83), RMSE of 5.68 h (95% CI: 4.65–6.71), MAPE of 14.55% (95% CI: 12.05–17.05), and R^2^ of 0.892 (95% CI: 0.850–0.934) (Table 1).

**Table 1:** Cross-validated performance of liver and kidney XGBoost models using PMI-specific top-20 metabolites.

Organ	Validation scheme	R^2^	RMSE (h)	MSE (h^2^)	MAE (h)	MAPE (%)
Liver	Repeated 10-fold CV	0.823 [0.732, 0.914]	6.66 [5.66, 7.65]	56.41 [41.19, 71.62]	4.78 [4.01, 5.54]	16.30 [13.55, 19.05]
Kidney		0.892 [0.850, 0.934]	5.68 [4.65, 6.71]	45.09 [27.62, 62.56]	4.05 [3.27, 4.83]	14.55 [12.05, 17.05]
Liver	Leave-one-time-point-out CV	0.587	15.22	231.53	13.52	39.20
Kidney		0.592	15.12	228.75	13.64	40.99

Values in brackets are 95% CIs calculated from the 50 validation-fold estimates of repeated 10-fold cross-validation. Leave-one-time-point-out metrics calculated from predictions pooled across the six held-out PMI levels. Samples at 0 h were excluded from MAPE calculation.

When each PMI level was withheld from feature selection and model training in turn, prediction errors increased in both organs. The liver model yielded an MAE of 13.52 h, RMSE of 15.22 h, MAPE of 39.20%, and R^2^ of 0.587, whereas the kidney model yielded an MAE of 13.64 h, RMSE of 15.12 h, MAPE of 40.99%, and R^2^ of 0.592 (Table 1). By comparison, under repeated 10-fold cross-validation, the corresponding MAE values were 4.78 h in liver and 4.05 h in kidney, and the MAPE values were 16.30% and 14.55%, respectively. Although the kidney model performed better numerically under repeated 10-fold cross-validation, this apparent advantage was not retained under leave-one-time-point-out validation, where its MAE and MAPE were slightly higher than those of the liver model.

**Fig 5:**
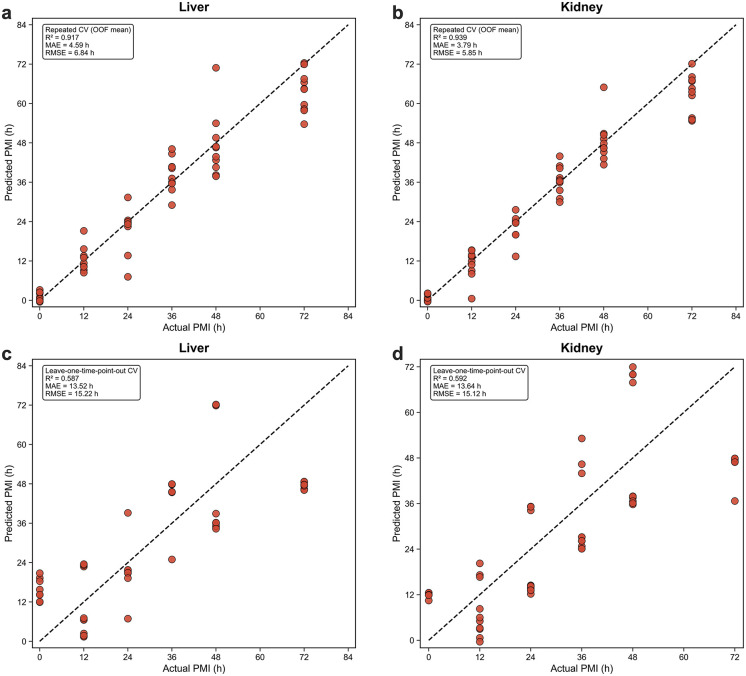
Prediction results of the PMI-specific top-20 XGBoost regression models. (a, b) Actual-versus-predicted PMI plots based on mean sample-level OOF predictions from repeated 10-fold cross-validation for liver (a) and kidney (b). Each sample contributed one OOF prediction per repeat, and the five predictions were averaged for plotting. (c, d) Actual-versus-predicted PMI plots from leave-one-time-point-out cross-validation for liver (c) and kidney (d). R^2^ values in panels a and b were calculated from the mean sample-level OOF predictions and are graphical summaries rather than the fold-level repeated-cross-validation estimates reported in Table 1. PMI, postmortem interval; OOF, out-of-fold; MAE, mean absolute error; RMSE, root mean squared error.

Mean OOF predictions from repeated 10-fold cross-validation are shown in Fig 5a,b, whereas leave-one-time-point-out predictions are shown in Fig 5c,d. The R^2^ values displayed in Fig 5a,b were calculated from the mean sample-level OOF predictions and were used only to visualize prediction trends; the formal repeated-cross-validation estimates are the fold-level values reported in Table 1. Under leave-one-time-point-out validation, the liver model overestimated 0 h samples by a mean of 15.29 h and underestimated 72 h samples by 24.43 h. The corresponding errors for the kidney model were +11.99 h at 0 h and −25.53 h at 72 h (S7 Table).

**Fig 6:**
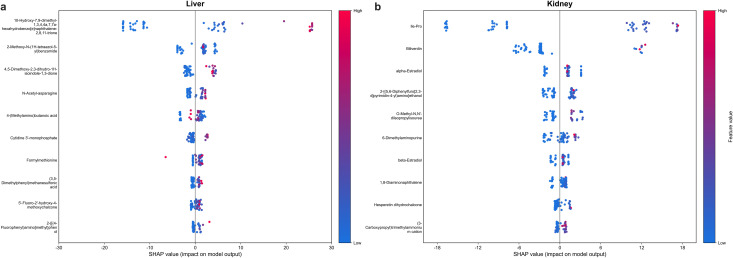
TreeSHAP feature-contribution summaries from the final-refit PMI-specific XGBoost models. (a, b) TreeSHAP beeswarm plots of the top 10 metabolites in the liver (a) and kidney (b) models. The organ-specific models were refitted using all samples and the global PMI-specific top-20 metabolites. Metabolites are ordered by mean absolute TreeSHAP value. Each point represents one sample, and colour indicates the relative metabolite value from low (blue) to high (pink). These panels are descriptive final-refit summaries and were not used to estimate predictive performance; fold-level TreeSHAP stability is reported in S10 Table.

TreeSHAP summaries from the final-refit models identified different leading features in liver and kidney. In liver, 10-Hydroxy-7,9-dimethyl-1,3,4,4a,7,7a-hexahydrobenzo[e]naphthalene-2,8,11-trione had the largest mean absolute TreeSHAP value (Fig 6a). In kidney, Ile-Pro and Biliverdin were among the leading contributors (Fig 6b). Gain-based importance, mean absolute TreeSHAP values, and TreeSHAP dependence plots from the final-refit models are shown in S5, S7, and S8 Figs., respectively.

Across repeated cross-validation, 10 liver metabolites and 7 kidney metabolites were included in the fold-specific top-20 feature sets in all 50 training folds (S6 Fig; S9 Table). Fold-level TreeSHAP summaries for the 20 highest-ranked metabolites in each organ, including their mean absolute contributions, mean ranks, and top-three and top-five frequencies, are reported in S10 Table. In the full-data Spearman analysis, ρ ranged from 0.939 to 0.962 for the liver top-20 metabolites and from 0.936 to 0.965 for the kidney top-20 metabolites; all FDR-adjusted P values were <0.001 (S8 Table).

## Discussion

In this controlled rat model, liver and kidney metabolomic profiles captured both differences between seawater drowning and postmortem submersion after CO_2_ euthanasia and variation associated with PMI. Classification performance remained high under repeated cross-validation, with mean AUCs of 0.973–0.996 in liver and 0.931–1.000 in kidney; after removal of PMI-specific mean shifts, the corresponding ranges were 0.980–0.987 and 0.936–0.993. In contrast, PMI regression performance decreased markedly when entire PMI levels were withheld, with MAE increasing from 4.78 to 13.52 h in liver and from 4.05 to 13.64 h in kidney. TreeSHAP and feature-selection analyses identified model-associated contribution and selection patterns, but these results were descriptive and did not establish the metabolites as validated biomarkers. Neither the predictive models nor the candidate metabolites were independently validated in the present study.

### Metabolic profiles of the liver and kidney tissues in the experimental model

Organic heterocyclic compounds, organic acids and derivatives, and lipids and lipid-like molecules were the three most frequently represented superclasses in both organs, with between-organ differences of only 0.3–1.4 percentage points. Because these proportions were based on annotation counts, they do not indicate relative metabolite abundance or metabolic activity. Annotation-confidence distributions were also comparable between organs. Level 2 assignments accounted for 73.0% of liver annotations and 74.3% of kidney annotations, whereas Level 1 assignments accounted for 20.9% and 19.3%, respectively; the remaining 6.1% and 6.5% were Level 3 assignments. Individual compounds assigned at Levels 2 and 3, particularly those classified as Levels 3.1 and 3.2, should therefore be regarded as putative until confirmed using authentic standards [21].

PCA indicated that PMI was the dominant source of overall metabolic variation. PC1 and PC2 together explained 51.9% of the variance in liver and 52.8% in kidney, and the corresponding score plots showed substantial overlap between D and PS samples; neither component showed an FDR-significant D/PS group effect. Significant group effects were instead detected in liver PC4, PC6, and PC7 and in kidney PC3, PC5, PC6, and PC7, whereas significant group × PMI interactions were confined to kidney PC5, PC7, and PC9. Thus, the D/PS difference was distributed across higher-order components rather than driving the dominant PC1–PC2 pattern. OPLS-DA showed group-associated structure in both organs, but visual separation and high fitted R^2^Y values alone were insufficient to demonstrate reproducible discrimination in this high-dimensional, small-sample dataset. Cross-validated OPLS-DA yielded Q^2^(cum) values of 0.847 in liver and 0.723 in kidney, with significant CV-ANOVA results and negative Q^2^ intercepts. However, the high permutation R^2^ intercepts of 0.880 and 0.876 showed that models fitted to permuted labels could still attain substantial apparent fit. Accordingly, the OPLS-DA results were interpreted as group-associated structure within the present dataset rather than as independent proof of a drowning-specific metabolic signature.

At 0 h, 54 liver and 61 kidney annotated metabolites met the differential-abundance criteria, but only nine liver metabolites and five kidney metabolites mapped to at least one KEGG pathway. The D0h–PS0h differences may reflect terminal physiological differences between the groups, including hypoxia and osmotic disturbance during drowning, but these mechanisms were not directly measured in the present study [4]. In liver, the pentose phosphate pathway was the only KEGG pathway containing more than one mapped differential metabolite; D-ribose and 2-deoxyribose 5-phosphate were both lower in D0h than in PS0h. Most remaining liver pathway entries were supported by a single mapped metabolite, and the mapped metabolites showed mixed directions of change. In kidney, five mapped metabolites generated 24 pathway entries because several compounds, particularly adenosine and dopamine, were mapped to multiple pathways. Adenosine and dopamine were higher in D0h than in PS0h, whereas 1-methylnicotinamide was lower. Therefore, the 16 liver and 24 kidney pathway entries should not be interpreted as equivalent numbers of independent pathway alterations or as evidence of pathway activation or inhibition. Accordingly, these findings are best interpreted as compound-level differences between the two experimental conditions at 0 h rather than as evidence of a coordinated drowning-specific pathway response.

Interpretation of the D0h–PS0h comparison is constrained by the control model. The PS group was generated by CO_2_ euthanasia before submersion, providing a standardized non-drowning control but not representing the range of deaths encountered in forensic casework. CO_2_ exposure can produce hypercapnia and respiratory acidosis before death and may therefore alter the baseline metabolic profile of the PS group [26]. Other euthanasia methods or death mechanisms, including pentobarbital administration, may produce different terminal physiological and metabolic conditions. Accordingly, the differential metabolites should be interpreted only as features distinguishing D0h from PS0h under this specific experimental design, rather than as biomarkers specific to drowning or as findings generalizable to all non-drowning deaths.

### Machine-learning discrimination and PMI regression using metabolomic profiles

Under repeated 10-fold cross-validation, mean AUCs ranged from 0.973 to 0.996 in liver and from 0.931 to 1.000 in kidney. RF had the highest mean AUC in liver (0.996), whereas GBDT had the highest mean AUC in kidney (1.000). These results show strong internal discrimination between D and PS samples within the present dataset, but performance in an independent cohort was not assessed. After PMI-specific mean shifts were removed within the training folds, mean AUCs remained 0.980–0.987 in liver and 0.936–0.993 in kidney. The classification results were therefore not attributable solely to average metabolic differences among the six PMI levels. Feature-selection stability was limited to a subset of metabolites: four liver metabolites and two kidney metabolites were included in the RF top-20 in all 50 folds, whereas the remaining features were selected less consistently. Previous studies have also applied supervised learning to postmortem metabolomic data for drowning-related discrimination and multi-organ PMI estimation [12,14]. Postmortem metabolomics has also been evaluated for large-scale cause-of-death screening in human cases [27] and PMI estimation across multiple tissues in an animal model [28]. A recent human femoral-blood study further demonstrated independent cross-platform validation of a metabolomics-based PMI model [29].

The performance of the PMI-specific XGBoost models differed substantially between the two validation schemes. Under repeated 10-fold cross-validation, the liver and kidney models achieved MAEs of 4.78 and 4.05 h and R^2^ values of 0.823 and 0.892, respectively. When entire PMI levels were withheld, the corresponding MAEs increased to 13.52 and 13.64 h, MAPE values increased to 39.20% and 40.99%, and R^2^ values decreased to 0.587 and 0.592. This discrepancy suggests that the repeated-cross-validation estimates may partly reflect prediction among samples from PMI levels already represented in the training data. Although the kidney model had a lower MAE and higher R^2^ than the liver model under repeated cross-validation, this apparent advantage was not retained under leave-one-time-point-out validation. The kidney model showed an MAE of 13.64 h, a MAPE of 40.99%, and a mean underestimation of 25.53 h at 72 h. These results do not support a claim of stable forensic utility for the kidney model. The liver model also remained exploratory, but the kidney model should not be favored on the basis of its repeated-cross-validation performance alone.

Repeated 10-fold cross-validation and leave-one-time-point-out validation address different prediction settings. The former evaluates samples from PMI levels represented in the training data, whereas the latter evaluates an entire PMI level excluded from both feature selection and model training. The higher errors under leave-one-time-point-out validation therefore indicate limited generalization to PMI levels absent from training. PMI-wise bias analysis showed that predictions at the temporal boundaries shifted toward the middle of the observed PMI range: 0 h was overestimated by 15.29 h in liver and 11.99 h in kidney, whereas 72 h was underestimated by 24.43 and 25.53 h, respectively (S7 Table). Thus, the models captured the broad temporal progression across the sampled 0–72 h interval but did not reliably estimate PMI levels omitted from training. The boundary errors in both organs also show that the lower repeated-cross-validation errors should not be interpreted as evidence of accurate prediction at unseen time points.

The TreeSHAP results from the final-refit models were descriptive and did not provide an additional estimate of predictive performance. In the final-refit liver model, 10-Hydroxy-7,9-dimethyl-1,3,4,4a,7,7a-hexahydrobenzo[e]naphthalene-2,8,11-trione had the largest mean absolute TreeSHAP value, whereas Ile-Pro and Biliverdin were among the leading features in the kidney model (Fig 6). The difference in leading features indicates that the two organ-specific models used different combinations of predictors, but it does not establish distinct biological mechanisms. Gain importance, mean absolute TreeSHAP values, and dependence plots from the final-refit models are shown in S5, S7, and S8 Figs., respectively. Across repeated cross-validation, 10 liver metabolites and 7 kidney metabolites were included in the fold-specific top-20 feature sets in all 50 training folds, while complete selection-frequency and fold-level TreeSHAP results are reported in S6 Fig. and S9–S10 Tables. In the full-data analysis, Spearman ρ ranged from 0.939 to 0.962 for the liver top-20 metabolites and from 0.936 to 0.965 for the kidney top-20 metabolites (S8 Table). Because these metabolites were selected using the same full-data correlation ranking, these coefficients describe the selection process rather than providing independent confirmation of their association with PMI. TreeSHAP contributions and selection frequencies therefore identify model-associated features, not causal metabolites or validated PMI biomarkers. Their chemical identities and biological relevance require targeted confirmation.

Several limitations define the scope of these findings. The study included only young male rats, with five animals per group at each of six fixed PMI levels; this design did not represent sex-, age-, or interindividual variation encountered in human cases. The study used only one non-drowning control condition—CO_2_ euthanasia followed by submersion—so the discriminative features cannot be assumed to distinguish drowning from other causes of death. All classification and PMI models were evaluated only within the present dataset. Neither model type was tested in an independent cohort, and the liver and kidney PMI models both had MAEs greater than 13 h when entire PMI levels were withheld. In particular, the kidney model retained a MAPE of 40.99% and underestimated the 72 h samples by a mean of 25.53 h, further limiting its current forensic applicability. All carcasses were examined using seawater from a single source under fixed temperature and humidity, so the effects of variation in seawater composition and postmortem environment were not evaluated. Postmortem metabolite patterns can vary with cause of death and ambient temperature [30,31]. Recent multi-organ research across different temperatures further indicates that organ-specific PMI models should be evaluated under variable environmental conditions [32]. Most annotations were assigned at Level 2 or Level 3, and the model-associated metabolites were not confirmed by targeted quantitative analysis using authentic standards.

Because the number of metabolite features greatly exceeded the number of animals in this study, model estimates were sensitive to feature selection and validation design [33]. Standardization, PMI-specific feature selection, and model fitting were therefore confined to the training folds to reduce information leakage. However, the divergence between repeated 10-fold and leave-one-time-point-out results shows that controlling information leakage did not establish generalization to PMI levels absent from training. Feature-selection frequencies, gain importance, and TreeSHAP summaries describe feature behavior within the present dataset but do not substitute for independent validation.

Further studies should include larger and more diverse cohorts, additional causes of death, independent validation sets, intermediate and later PMI levels, variable postmortem environments, and targeted confirmation of candidate metabolites; evaluation in human forensic material will also be necessary.

## Supporting information

S1 Fig Principal component analysis (PCA) plots including quality control (QC) samples. (a, b) PCA score plots of liver (a) and kidney (b) metabolomic datasets with QC samples included. Tight clustering of QC samples indicates good analytical reproducibility.
(TIFF)

S2 Fig Performance of PMI-adjusted classification models and stability of RF-selected top-20 metabolites. (a, b) Mean receiver operating characteristic (ROC) curves with 95% confidence intervals (CIs) for the PMI-adjusted sensitivity analysis in liver (a) and kidney (b). PMI-specific mean shifts for each metabolite were removed within the training folds and the corresponding adjustment was applied to the validation folds before model retraining under the same repeated 10-fold cross-validation framework. (c, d) Top-20 selection frequency of RF-ranked metabolites across repeated cross-validation iterations in liver (c) and kidney (d), based on the primary classification analysis.
(TIFF)

S3 Fig Confusion matrices of four classification models in the liver metabolomic dataset. Using the same repeated 10-fold cross-validation framework as in the main analysis, confusion matrices were generated for four classification models to distinguish the seawater drowning group (D) from the postmortem submersion group (PS) in the liver metabolomic dataset. (a) Random Forest (RF); (b) Support Vector Machine (SVM); (c) Multi-Layer Perceptron (MLP); and (d) Gradient Boosting Decision Tree (GBDT). Each matrix shows the agreement between true and predicted class labels, including true negatives (TN), false positives (FP), false negatives (FN), and true positives (TP).
(TIFF)

S4 Fig Confusion matrices of four classification models in the kidney metabolomic dataset. Using the same repeated 10-fold cross-validation framework as in the main analysis, confusion matrices were generated for four classification models to distinguish the seawater drowning group (D) from the postmortem submersion group (PS) in the kidney metabolomic dataset. (a) Random Forest (RF); (b) Support Vector Machine (SVM); (c) Multi-Layer Perceptron (MLP); and (d) Gradient Boosting Decision Tree (GBDT). Each matrix shows the agreement between true and predicted class labels, including true negatives (TN), false positives (FP), false negatives (FN), and true positives (TP).
(TIFF)

S5 Fig Relative XGBoost gain importance of PMI-specific metabolites. (a) Liver; (b) kidney. Bars show the relative gain importance of the top 20 metabolites in the final organ-specific XGBoost models.
(TIFF)

S6 Fig Selection frequency of PMI-specific Top20 metabolites across repeated cross-validation folds. (a) Liver; (b) kidney. Bars show the proportion of the 50 training folds in which each metabolite was included in the fold-specific top-20 feature set.
(TIFF)

S7 Fig Mean absolute SHAP values of PMI-specific XGBoost regression models. (a) Liver; (b) kidney. Bars show the mean absolute TreeSHAP values of the top 20 metabolites in the final organ-specific XGBoost models.
(TIFF)

S8 Fig SHAP dependence plots for the top six metabolites in PMI-specific XGBoost regression models. (a) Liver; (b) kidney. Dependence plots are shown for the six metabolites with the highest mean absolute TreeSHAP values in each organ. Each point represents one sample; the x-axis shows the metabolite feature value, and the y-axis shows the corresponding TreeSHAP value.
(TIFF)

S1 Table Explained variance and effects of D/PS group, PMI, and their interaction across the first ten principal components. **Note:** PCA scores were generated using SIMCA. The effects of D/PS group, PMI, and their interaction were assessed separately for each principal component using two-factor linear models. PMI was treated as a categorical variable. FDR values were adjusted using the Benjamini–Hochberg method across PC1–PC10 separately for each organ and each effect. Bold values indicate FDR < 0.05. Partial η^2^ represents the effect size of the overall D/PS group effect after accounting for PMI and the Group × PMI interaction. D, drowning; PS, postmortem submersion; PMI, postmortem interval.
(DOCX)

S2 Table Internal validation summary of the five-fold cross-validated OPLS-DA models. R^2^X(cum), cumulative proportion of explained X variance; R^2^Y(cum), cumulative proportion of explained class-label variance; Q^2^(cum), cumulative cross-validated predictive ability. CV-ANOVA was based on cross-validated residuals. R^2^ and Q^2^ intercepts were obtained from 200-response permutation tests.
(DOCX)

S3 Table KEGG pathway mapping summary of differential metabolites identified in the D0h versus PS0h comparison. Differential metabolites were defined as those meeting both FDR < 0.05 and |log₂FC| > 1. The table lists KEGG pathway entries containing at least one mapped differential metabolite, together with the mapped metabolite names, KEGG compound IDs, annotation levels, and the direction of each mapped metabolite in the D0h versus PS0h comparison. The reported direction refers to individual mapped metabolites and does not indicate pathway-level upregulation or downregulation. Annotation level indicates the relative confidence of metabolite identification as reported by the metabolite annotation workflow.
(XLSX)

S4 Table Performance metrics of four machine learning classifiers for cause-of-death discrimination in liver and kidney metabolomic datasets under repeated 10-fold cross-validation (five repeats; 50 folds total). Metrics are presented as mean ± standard deviation.
(DOCX)

S5 Table Performance metrics of the PMI-adjusted sensitivity analysis for four machine learning classifiers in liver and kidney metabolomic datasets under repeated 10-fold cross-validation (five repeats; 50 folds total). Metrics are presented as mean ± standard deviation; AUC values are additionally summarized with 95% confidence intervals.
(DOCX)

S6 Table Stability summary of RF-selected top-20 metabolites across repeated cross-validation iterations in the primary classification analysis. For each organ, the table summarizes feature importance (mean ± SD), rank (mean ± SD), and top-20 selection frequency.
(DOCX)

S7 Table PMI-wise prediction bias of PMI-specific Top20 XGBoost regression models. Predicted PMI and prediction error are shown as mean ± SD, whereas absolute error is shown as mean value.
(DOCX)

S8 Table Spearman correlations between PMI and the global top-20 metabolites in liver and kidney. PMI, postmortem interval; FDR, false discovery rate. P values and FDR-adjusted P values are reported as <0.001 for readability.
(DOCX)

S9 Table Selection frequency of PMI-specific metabolites across repeated cross-validation folds. The table lists the 20 most frequently selected metabolites in liver and kidney across 50 training folds.
(DOCX)

S10 Table Fold-level TreeSHAP stability of PMI-specific metabolites. The table lists the 20 metabolites with the highest mean absolute TreeSHAP values in liver and kidney, together with their mean TreeSHAP ranks and top-three and top-five frequencies.
(DOCX)

S11 Table Distribution of metabolite annotation confidence levels in the liver and kidney datasets. (DOCX)
