# Supplementary figures and images for: Integrated machine learning for cause-of-death classification and postmortem interval prediction: Liver and kidney metabolomics from seawater-immersed rat cadavers
Source: PLoS One. 2026 Jul 23;21(7):e0353958. doi: 10.1371/journal.pone.0353958 (PMC13395348; doi:10.1371/journal.pone.0353958)

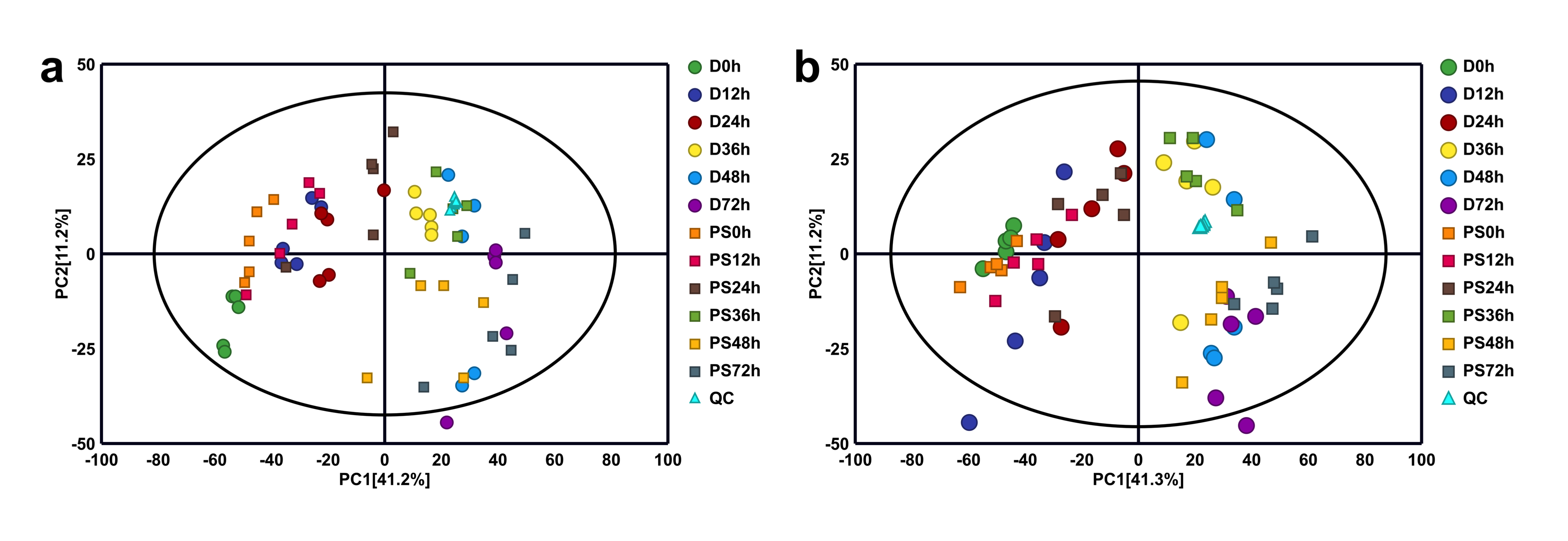

Supplement: S1 Fig — (a, b) PCA score plots of liver (a) and kidney (b) metabolomic datasets with QC samples included. Tight clustering of QC samples indicates good analytical reproducibility. (TIFF) [file pone.0353958.s001.tiff]

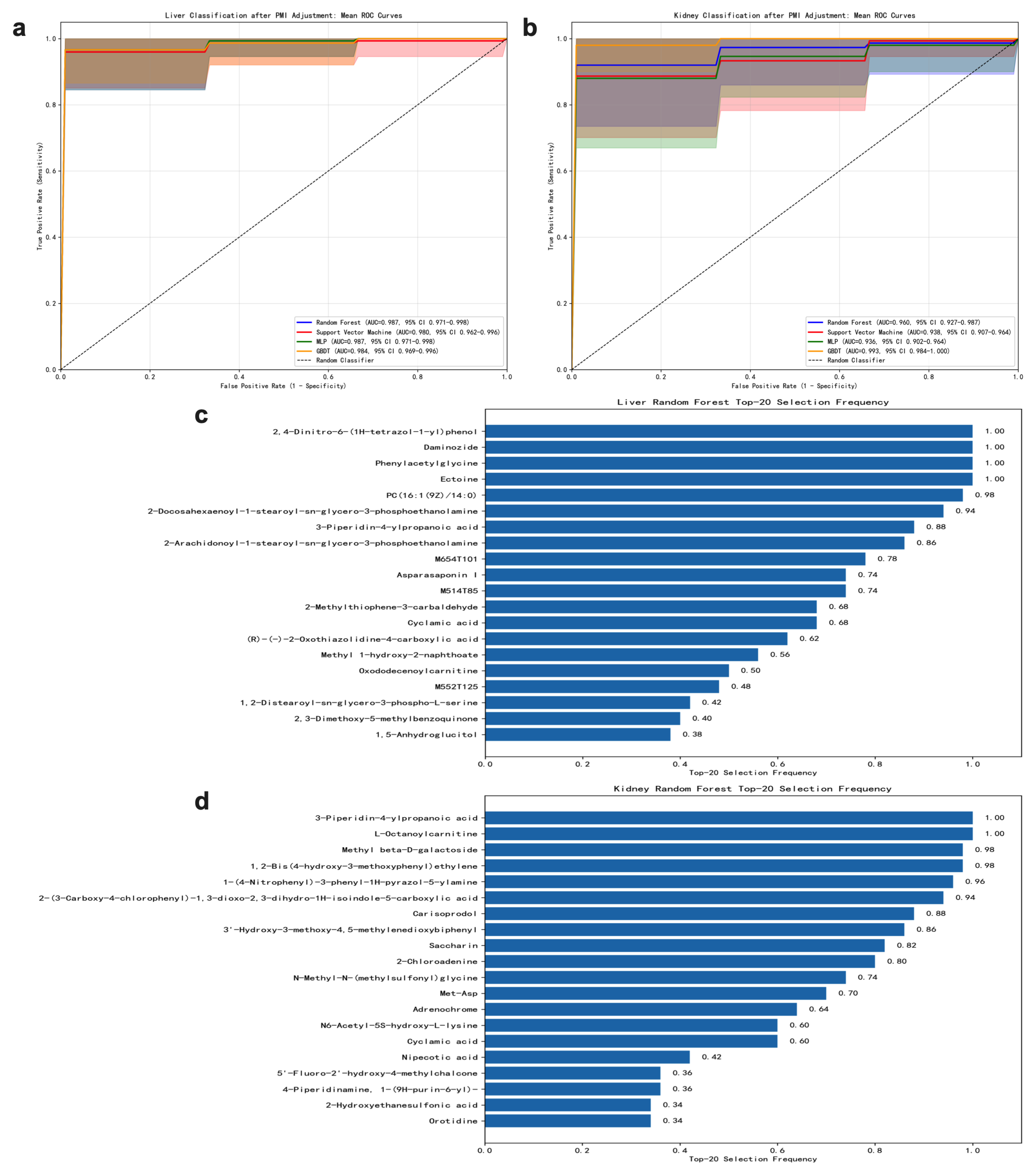

Supplement: S2 Fig — (a, b) Mean receiver operating characteristic (ROC) curves with 95% confidence intervals (CIs) for the PMI-adjusted sensitivity analysis in liver (a) and kidney (b). PMI-specific mean shifts for each metabolite were removed within the training folds and the corresponding adjustment was applied to the validation folds before model retraining under the same repeated 10-fold cross-validation framework. (c, d) Top-20 selection frequency of RF-ranked metabolites across repeated cross-validation iterations in liver (c) and kidney (d), based on the primary classification analysis. (TIFF) [file pone.0353958.s002.tiff]

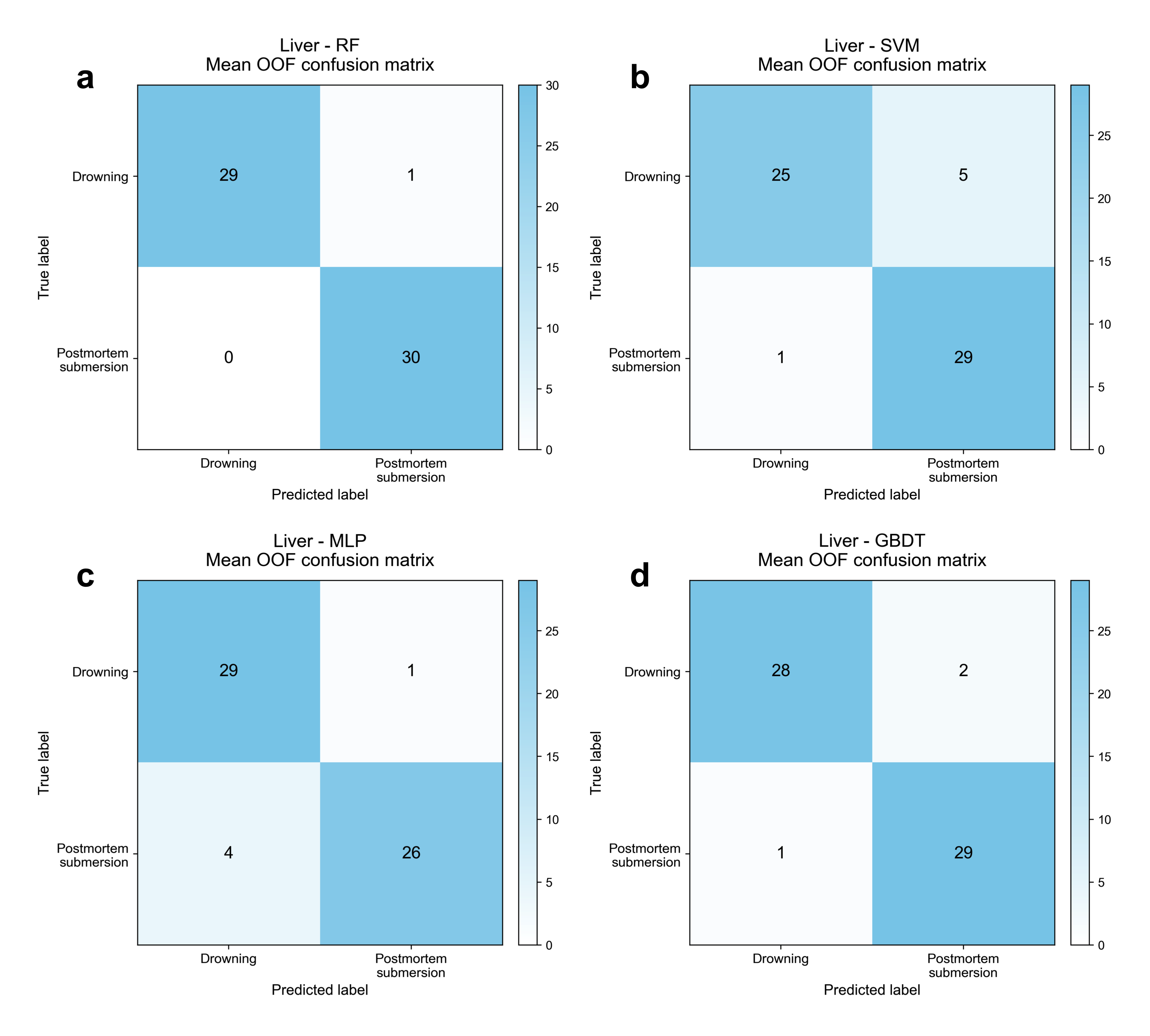

Supplement: S3 Fig — Using the same repeated 10-fold cross-validation framework as in the main analysis, confusion matrices were generated for four classification models to distinguish the seawater drowning group (D) from the postmortem submersion group (PS) in the liver metabolomic dataset. (a) Random Forest (RF); (b) Support Vector Machine (SVM); (c) Multi-Layer Perceptron (MLP); and (d) Gradient Boosting Decision Tree (GBDT). Each matrix shows the agreement between true and predicted class labels, including true negatives (TN), false positives (FP), false negatives (FN), and true positives (TP). (TIFF) [file pone.0353958.s003.tiff]

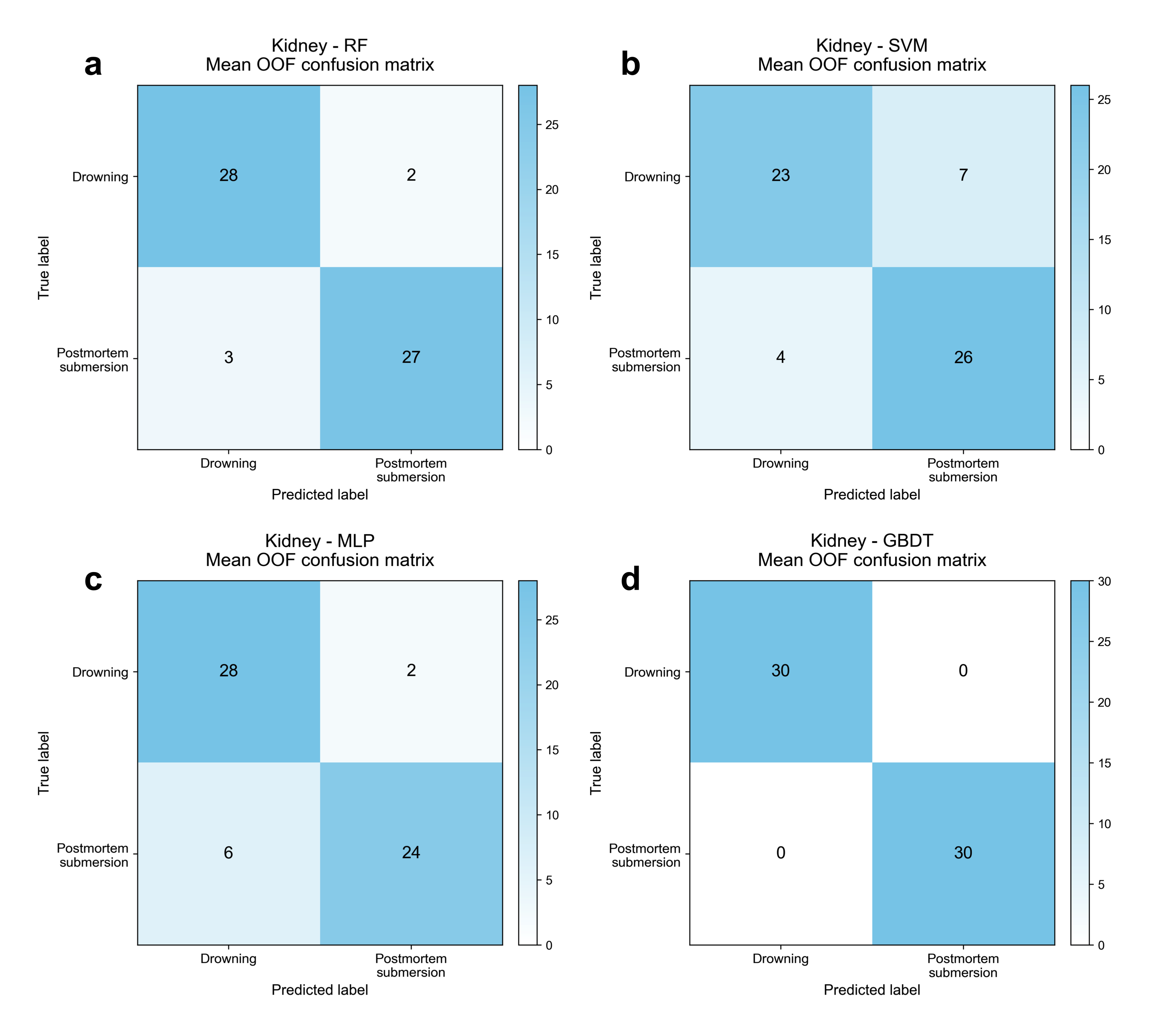

Supplement: S4 Fig — Using the same repeated 10-fold cross-validation framework as in the main analysis, confusion matrices were generated for four classification models to distinguish the seawater drowning group (D) from the postmortem submersion group (PS) in the kidney metabolomic dataset. (a) Random Forest (RF); (b) Support Vector Machine (SVM); (c) Multi-Layer Perceptron (MLP); and (d) Gradient Boosting Decision Tree (GBDT). Each matrix shows the agreement between true and predicted class labels, including true negatives (TN), false positives (FP), false negatives (FN), and true positives (TP). (TIFF) [file pone.0353958.s004.tiff]

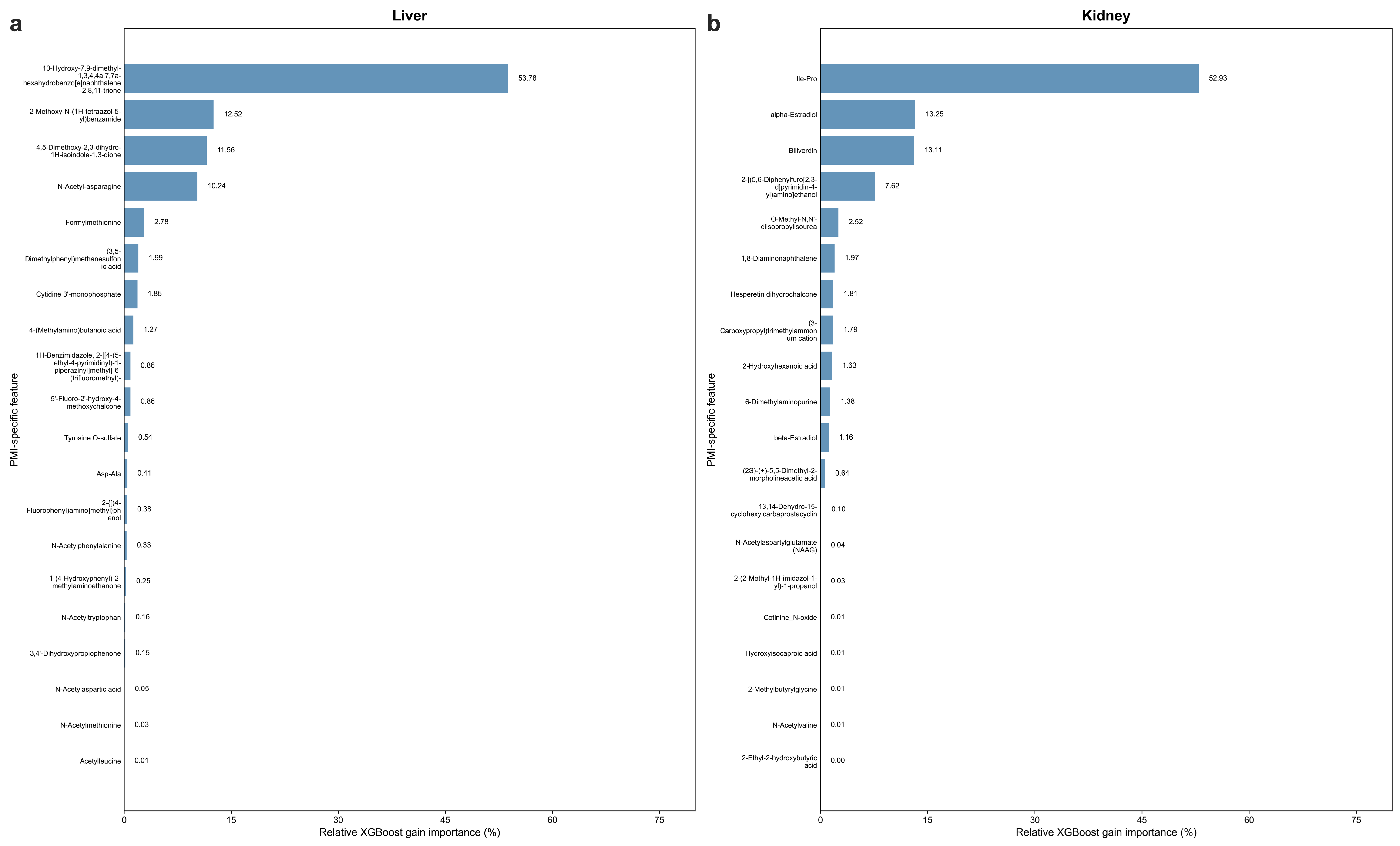

Supplement: S5 Fig — (a) Liver; (b) kidney. Bars show the relative gain importance of the top 20 metabolites in the final organ-specific XGBoost models. (TIFF) [file pone.0353958.s005.tiff]

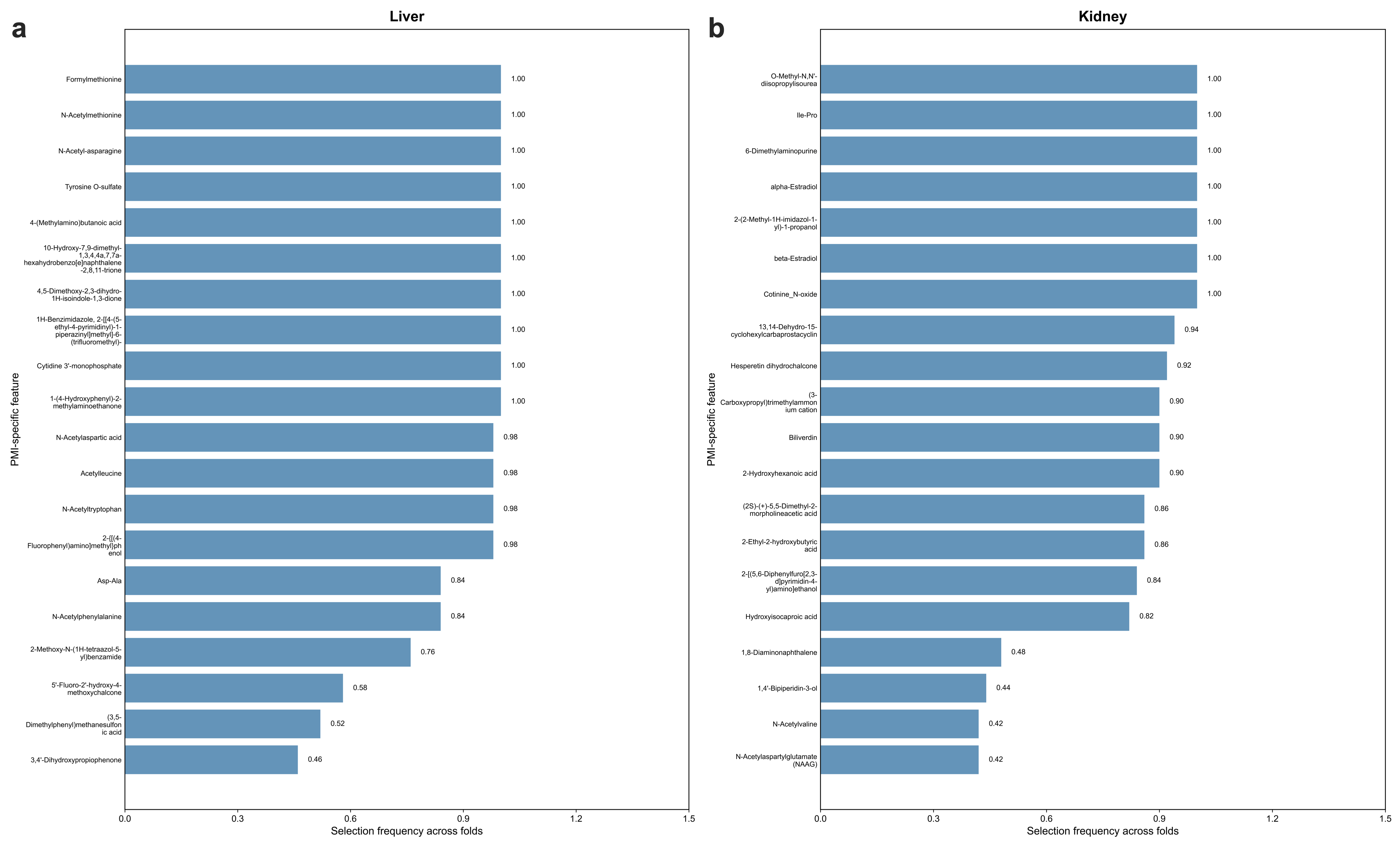

Supplement: S6 Fig — (a) Liver; (b) kidney. Bars show the proportion of the 50 training folds in which each metabolite was included in the fold-specific top-20 feature set. (TIFF) [file pone.0353958.s006.tiff]

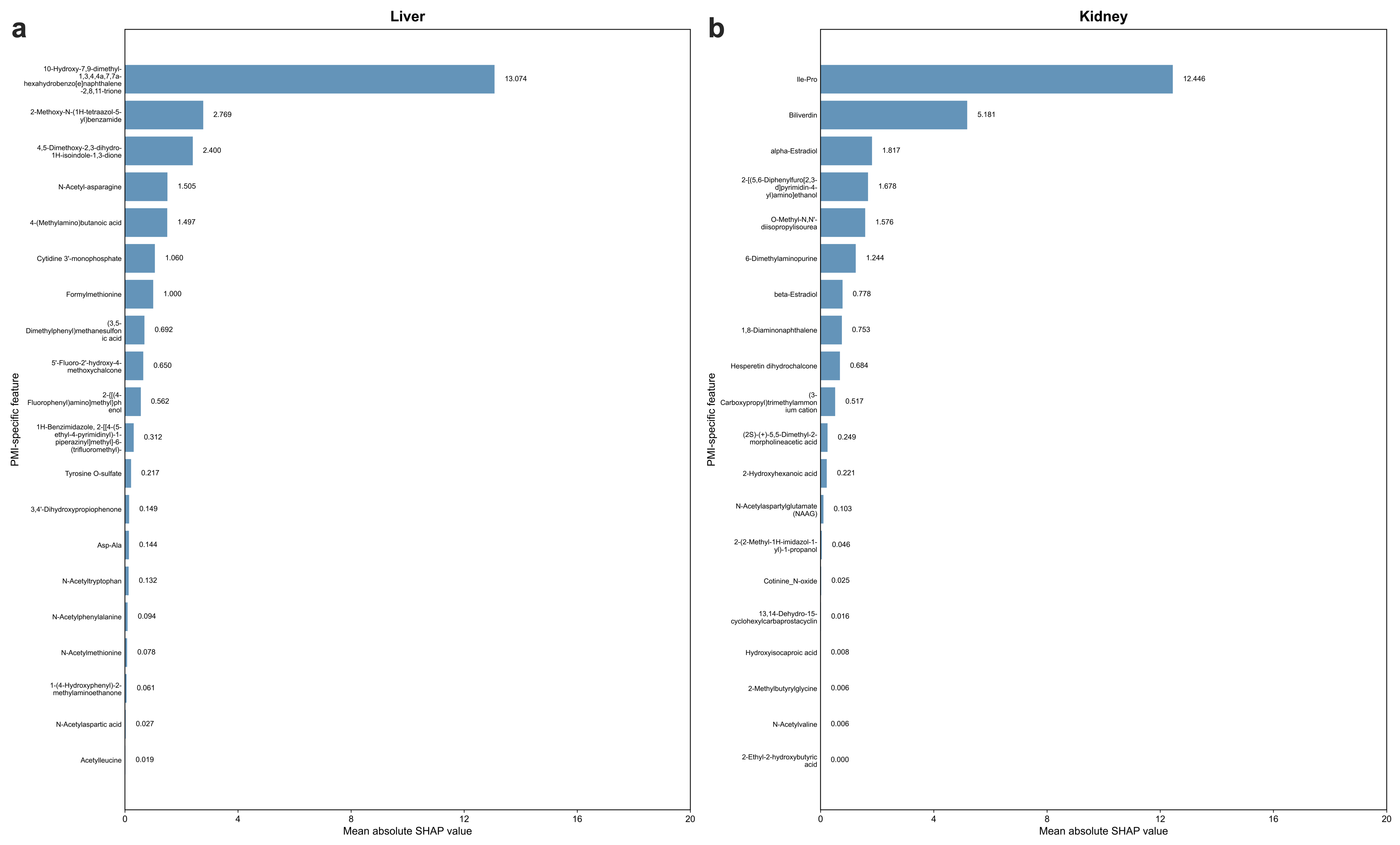

Supplement: S7 Fig — (a) Liver; (b) kidney. Bars show the mean absolute TreeSHAP values of the top 20 metabolites in the final organ-specific XGBoost models. (TIFF) [file pone.0353958.s007.tiff]

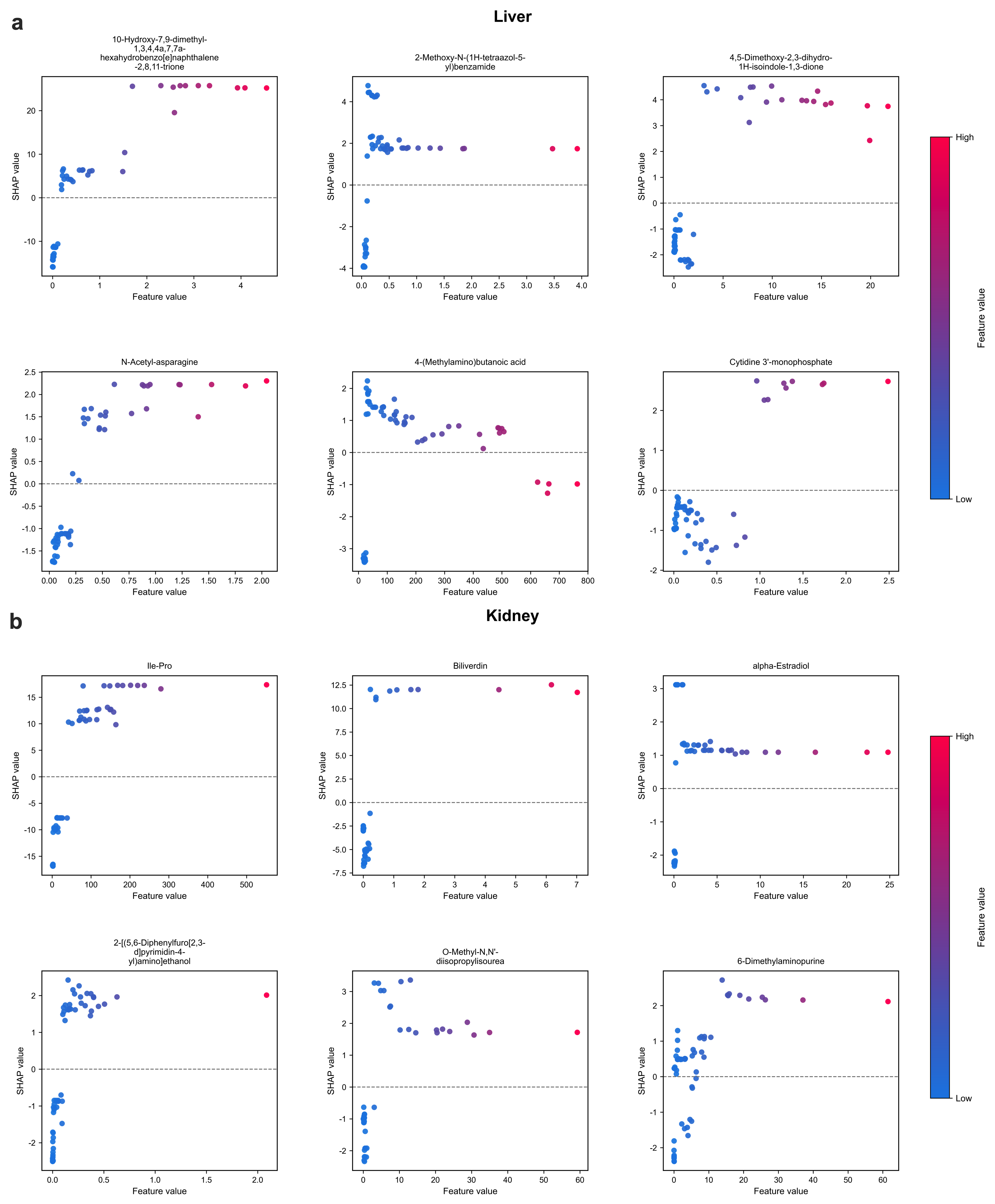

Supplement: S8 Fig — (a) Liver; (b) kidney. Dependence plots are shown for the six metabolites with the highest mean absolute TreeSHAP values in each organ. Each point represents one sample; the x-axis shows the metabolite feature value, and the y-axis shows the corresponding TreeSHAP value. (TIFF) [file pone.0353958.s008.tiff]
