# Supplementary material for: Integrated machine learning for cause-of-death classification and postmortem interval prediction: Liver and kidney metabolomics from seawater-immersed rat cadavers
Source: PLoS One. 2026 Jul 23;21(7):e0353958. doi: 10.1371/journal.pone.0353958 (PMC13395348; doi:10.1371/journal.pone.0353958)
Supplement: S1 Table — Note: PCA scores were generated using SIMCA. The effects of D/PS group, PMI, and their interaction were assessed separately for each principal component using two-factor linear models. PMI was treated as a categorical variable. FDR values were adjusted using the Benjamini–Hochberg method across PC1–PC10 separately for each organ and each effect. Bold values indicate FDR < 0.05. Partial η2 represents the effect size of the overall D/PS group effect after accounting for PMI and the Group × PMI interaction. D, drowning; PS, postmortem submersion; PMI, postmortem interval. (DOCX) [file pone.0353958.s009.docx]

**S1 Table. Explained variance and effects of D/PS group, PMI, and their interaction across the first ten principal components.**

| **Organ** | **Principal component** | **Explained variance (%)** | **Cumulative variance (%)** | **Group effect FDR** | **Group partial η^2^** | **PMI effect FDR** | **Group × PMI FDR** |
| --- | --- | --- | --- | --- | --- | --- | --- |
| Liver | PC1 | 40.90 | 40.90 | 0.640 | 0.012 | 1.39 × 10⁻²⁶ | 0.294 |
|  | PC2 | 11.00 | 51.90 | 0.715 | 0.006 | 1.99 × 10⁻⁴ | 0.190 |
|  | PC3 | 5.95 | 57.90 | 0.229 | 0.058 | 1.53 × 10⁻⁵ | 0.190 |
|  | PC4 | 4.81 | 62.70 | 3.63 × 10⁻¹⁰ | 0.602 | 0.002 | 0.068 |
|  | PC5 | 3.75 | 66.50 | 0.773 | 0.002 | 0.014 | 0.958 |
|  | PC6 | 3.27 | 69.70 | 0.029 | 0.135 | 0.022 | 0.068 |
|  | PC7 | 2.39 | 72.10 | 5.46 × 10⁻⁵ | 0.334 | 3.49 × 10⁻⁴ | 0.367 |
|  | PC8 | 2.22 | 74.30 | 0.715 | 0.005 | 0.033 | 0.860 |
|  | PC9 | 1.96 | 76.30 | 0.300 | 0.043 | 0.574 | 0.089 |
|  | PC10 | 1.51 | 77.80 | 0.404 | 0.028 | 0.723 | 0.067 |
| Kidney | PC1 | 41.20 | 41.20 | 0.374 | 0.031 | 3.37 × 10⁻²⁹ | 0.470 |
|  | PC2 | 11.50 | 52.80 | 0.390 | 0.025 | 4.37 × 10⁻⁴ | 0.470 |
|  | PC3 | 7.66 | 60.40 | 7.45 × 10⁻⁵ | 0.326 | 1.59 × 10⁻⁷ | 0.386 |
|  | PC4 | 3.39 | 63.80 | 0.447 | 0.018 | 0.016 | 0.727 |
|  | PC5 | 3.08 | 66.90 | 0.013 | 0.152 | 0.004 | **0.005** |
|  | PC6 | 2.65 | 69.60 | 2.17 × 10⁻⁷ | 0.483 | 2.47 × 10⁻¹¹ | 0.130 |
|  | PC7 | 2.20 | 71.70 | 0.012 | 0.162 | 0.004 | **0.005** |
|  | PC8 | 1.91 | 73.70 | 0.662 | 0.006 | 0.034 | 0.536 |
|  | PC9 | 1.82 | 75.50 | 0.072 | 0.088 | 0.577 | **0.005** |
|  | PC10 | 1.55 | 77.00 | 0.905 | <0.001 | 0.755 | 0.468 |

**Note:** PCA scores were generated using SIMCA. The effects of D/PS group, PMI, and their interaction were assessed separately for each principal component using two-factor linear models. PMI was treated as a categorical variable. FDR values were adjusted using the Benjamini–Hochberg method across PC1–PC10 separately for each organ and each effect. Bold values indicate FDR < 0.05. Partial η^2^ represents the effect size of the overall D/PS group effect after accounting for PMI and the Group × PMI interaction. D, drowning; PS, postmortem submersion; PMI, postmortem interval.
