# Supplementary material for: Integrated machine learning for cause-of-death classification and postmortem interval prediction: Liver and kidney metabolomics from seawater-immersed rat cadavers
Source: PLoS One. 2026 Jul 23;21(7):e0353958. doi: 10.1371/journal.pone.0353958 (PMC13395348; doi:10.1371/journal.pone.0353958)
Supplement: S2 Table — R2X(cum), cumulative proportion of explained X variance; R2Y(cum), cumulative proportion of explained class-label variance; Q2(cum), cumulative cross-validated predictive ability. CV-ANOVA was based on cross-validated residuals. R2 and Q2 intercepts were obtained from 200-response permutation tests. (DOCX) [file pone.0353958.s010.docx]

**S2 Table. Internal validation summary of the five-fold cross-validated OPLS-DA models.** R^2^X(cum), cumulative proportion of explained X variance; R^2^Y(cum), cumulative proportion of explained class-label variance; Q^2^(cum), cumulative cross-validated predictive ability. CV-ANOVA was based on cross-validated residuals. R^2^ and Q^2^ intercepts were obtained from 200-response permutation tests.

| **Organ** | **Components** | **R^2^X(cum)** | **R^2^Y(cum)** | **Q^2^(cum)** | **CV-ANOVA F** | **df** | **p value** | **R^2^ intercept** | **Q^2^ intercept** |
| --- | --- | --- | --- | --- | --- | --- | --- | --- | --- |
| Liver | 1+4+0 | 0.654 | 0.983 | 0.847 | 27.139 | 10, 49 | 1.23 × 10⁻¹⁶ | 0.880 | −0.448 |
| Kidney | 1+4+0 | 0.672 | 0.970 | 0.723 | 12.785 | 10, 49 | 1.41 × 10⁻¹⁰ | 0.876 | −0.378 |
