# Supplementary material for: Integrated machine learning for cause-of-death classification and postmortem interval prediction: Liver and kidney metabolomics from seawater-immersed rat cadavers
Source: PLoS One. 2026 Jul 23;21(7):e0353958. doi: 10.1371/journal.pone.0353958 (PMC13395348; doi:10.1371/journal.pone.0353958)
Supplement: S4 Table — Metrics are presented as mean ± standard deviation. (DOCX) [file pone.0353958.s012.docx]

**S4 Table. Performance metrics of four machine learning classifiers for cause-of-death discrimination in liver and kidney metabolomic datasets under repeated 10-fold cross-validation (five repeats; 50 folds total).** Metrics are presented as mean ± standard deviation.

| **Organ** | **Model** | **Accuracy** | **Precision** | **Recall** | **F1 score** | **AUC (mean ± SD)** | **AUC (95% CI)** |
| --- | --- | --- | --- | --- | --- | --- | --- |
| **Liver** | **RF** | 0.970 ± 0.065 | 0.975 ± 0.076 | 0.973 ± 0.091 | 0.970 ± 0.066 | 0.996 ± 0.031 | 0.987–1.000 |
|  | **SVM** | 0.893 ± 0.157 | 0.886 ± 0.171 | 0.960 ± 0.128 | 0.908 ± 0.134 | 0.980 ± 0.080 | 0.956–0.998 |
|  | **MLP** | 0.913 ± 0.108 | 0.970 ± 0.092 | 0.867 ± 0.190 | 0.900 ± 0.130 | 0.973 ± 0.073 | 0.951–0.991 |
|  | **GBDT** | 0.927 ± 0.117 | 0.935 ± 0.129 | 0.933 ± 0.165 | 0.923 ± 0.132 | 0.976 ± 0.085 | 0.951–0.996 |
| **Kidney** | **RF** | 0.920 ± 0.118 | 0.955 ± 0.109 | 0.893 ± 0.207 | 0.906 ± 0.152 | 0.984 ± 0.050 | 0.969–0.998 |
|  | **SVM** | 0.833 ± 0.165 | 0.867 ± 0.183 | 0.840 ± 0.226 | 0.828 ± 0.177 | 0.938 ± 0.117 | 0.902–0.967 |
|  | **MLP** | 0.870 ± 0.127 | 0.940 ± 0.119 | 0.813 ± 0.253 | 0.841 ± 0.177 | 0.931 ± 0.125 | 0.893–0.962 |
|  | **GBDT** | 0.993 ± 0.047 | 0.992 ± 0.057 | 1.000 ± 0.000 | 0.995 ± 0.035 | 1.000 ± 0.000 | 1.000–1.000 |
