# Supplementary material for: Integrated machine learning for cause-of-death classification and postmortem interval prediction: Liver and kidney metabolomics from seawater-immersed rat cadavers
Source: PLoS One. 2026 Jul 23;21(7):e0353958. doi: 10.1371/journal.pone.0353958 (PMC13395348; doi:10.1371/journal.pone.0353958)
Supplement: S5 Table — Metrics are presented as mean ± standard deviation; AUC values are additionally summarized with 95% confidence intervals. (DOCX) [file pone.0353958.s013.docx]

**S5 Table. Performance metrics of the PMI-adjusted sensitivity analysis for four machine learning classifiers in liver and kidney metabolomic datasets under repeated 10-fold cross-validation (five repeats; 50 folds total).** Metrics are presented as mean ± standard deviation; AUC values are additionally summarized with 95% confidence intervals.

| **Organ** | **Model** | **Accuracy** | **Precision** | **Recall** | **F1 score** | **AUC (mean ± SD)** | **AUC (95% CI)** |
| --- | --- | --- | --- | --- | --- | --- | --- |
| **Liver** | **RF** | 0.943 ± 0.104 | 0.963 ± 0.101 | 0.927 ± 0.139 | 0.940 ± 0.109 | 0.987 ± 0.048 | 0.971–0.998 |
|  | **SVM** | 0.880 ± 0.147 | 0.847 ± 0.171 | 0.993 ± 0.047 | 0.905 ± 0.111 | 0.980 ± 0.062 | 0.962–0.996 |
|  | **MLP** | 0.947 ± 0.103 | 0.960 ± 0.101 | 0.940 ± 0.146 | 0.943 ± 0.116 | 0.987 ± 0.048 | 0.971–0.998 |
|  | **GBDT** | 0.933 ± 0.112 | 0.957 ± 0.113 | 0.920 ± 0.144 | 0.931 ± 0.111 | 0.984 ± 0.050 | 0.969–0.996 |
| **Kidney** | **RF** | 0.910 ± 0.118 | 0.965 ± 0.101 | 0.860 ± 0.214 | 0.892 ± 0.153 | 0.960 ± 0.114 | 0.927–0.987 |
|  | **SVM** | 0.800 ± 0.187 | 0.842 ± 0.256 | 0.760 ± 0.309 | 0.762 ± 0.254 | 0.938 ± 0.108 | 0.907–0.964 |
|  | **MLP** | 0.853 ± 0.157 | 0.897 ± 0.169 | 0.813 ± 0.244 | 0.833 ± 0.190 | 0.936 ± 0.115 | 0.902–0.964 |
|  | **GBDT** | 0.950 ± 0.091 | 0.944 ± 0.116 | 0.980 ± 0.080 | 0.955 ± 0.079 | 0.993 ± 0.027 | 0.984–1.000 |
