# Supplementary material for: Integrated machine learning for cause-of-death classification and postmortem interval prediction: Liver and kidney metabolomics from seawater-immersed rat cadavers
Source: PLoS One. 2026 Jul 23;21(7):e0353958. doi: 10.1371/journal.pone.0353958 (PMC13395348; doi:10.1371/journal.pone.0353958)
Supplement: S6 Table — For each organ, the table summarizes feature importance (mean ± SD), rank (mean ± SD), and top-20 selection frequency. (DOCX) [file pone.0353958.s014.docx]

**S6 Table. Stability summary of RF-selected top-20 metabolites across repeated cross-validation iterations in the primary classification analysis.** For each organ, the table summarizes feature importance (mean ± SD), rank (mean ± SD), and top-20 selection frequency.

| **Organ** | **Feature** | **Feature importance (mean ± SD)** | **Rank (mean ± SD)** | **Top20 selection frequency** |
| --- | --- | --- | --- | --- |
| Liver | 2,4-Dinitro-6-(1H-tetrazol-1-yl)phenol | 0.0194 ± 0.0031 | 3.24 ± 2.63 | 1.00 |
|  | Daminozide | 0.0192 ± 0.0050 | 3.98 ± 3.69 | 1.00 |
|  | Phenylacetylglycine | 0.0172 ± 0.0026 | 4.44 ± 2.42 | 1.00 |
|  | Ectoine | 0.0130 ± 0.0022 | 9.82 ± 4.03 | 1.00 |
|  | PC(16:1(9Z)/14:0) | 0.0177 ± 0.0046 | 5.50 ± 5.97 | 0.98 |
|  | 2-Docosahexaenoyl-1-stearoyl-sn-glycero-3-phosphoethanolamine | 0.0153 ± 0.0040 | 7.16 ± 5.37 | 0.94 |
|  | 3-Piperidin-4-ylpropanoic acid | 0.0120 ± 0.0028 | 12.48 ± 6.31 | 0.88 |
|  | 2-Arachidonoyl-1-stearoyl-sn-glycero-3-phosphoethanolamine | 0.0136 ± 0.0047 | 14.82 ± 27.26 | 0.86 |
|  | M654T101 | 0.0110 ± 0.0034 | 15.98 ± 10.35 | 0.78 |
|  | Asparasaponin I | 0.0110 ± 0.0034 | 18.04 ± 16.32 | 0.74 |
|  | M514T85 | 0.0106 ± 0.0032 | 65.44 ± 345.06 | 0.74 |
|  | 2-Methylthiophene-3-carbaldehyde | 0.0108 ± 0.0053 | 23.12 ± 24.20 | 0.68 |
|  | Cyclamic acid | 0.0101 ± 0.0029 | 18.30 ± 10.44 | 0.68 |
|  | (R)-(-)-2-Oxothiazolidine-4-carboxylic acid | 0.0101 ± 0.0037 | 20.70 ± 14.77 | 0.62 |
|  | Methyl 1-hydroxy-2-naphthoate | 0.0094 ± 0.0049 | 38.36 ± 47.64 | 0.56 |
|  | Oxododecenoylcarnitine | 0.0096 ± 0.0048 | 31.34 ± 41.79 | 0.50 |
|  | M552T125 | 0.0085 ± 0.0029 | 26.66 ± 17.93 | 0.48 |
|  | 1,2-Distearoyl-sn-glycero-3-phospho-L-serine | 0.0083 ± 0.0029 | 30.46 ± 39.02 | 0.42 |
|  | 2,3-Dimethoxy-5-methylbenzoquinone | 0.0078 ± 0.0042 | 66.00 ± 206.88 | 0.40 |
|  | 1,5-Anhydroglucitol | 0.0077 ± 0.0042 | 109.58 ± 343.08 | 0.38 |
| Kidney | 3-Piperidin-4-ylpropanoic acid | 0.0383 ± 0.0022 | 1.00 ± 0.00 | 1.00 |
|  | L-Octanoylcarnitine | 0.0217 ± 0.0027 | 2.62 ± 0.83 | 1.00 |
|  | Methyl beta-D-galactoside | 0.0169 ± 0.0051 | 5.94 ± 6.25 | 0.98 |
|  | 1,2-Bis(4-hydroxy-3-methoxyphenyl)ethylene | 0.0130 ± 0.0032 | 9.10 ± 6.26 | 0.98 |
|  | 1-(4-Nitrophenyl)-3-phenyl-1H-pyrazol-5-ylamine | 0.0173 ± 0.0052 | 5.88 ± 6.72 | 0.96 |
|  | 2-(3-Carboxy-4-chlorophenyl)-1,3-dioxo-2,3-dihydro-1H-isoindole-5-carboxylic acid | 0.0106 ± 0.0024 | 12.90 ± 5.17 | 0.94 |
|  | Carisoprodol | 0.0126 ± 0.0042 | 12.30 ± 13.06 | 0.88 |
|  | 3'-Hydroxy-3-methoxy-4,5-methylenedioxybiphenyl | 0.0117 ± 0.0036 | 13.76 ± 18.91 | 0.86 |
|  | Saccharin | 0.0111 ± 0.0043 | 19.90 ± 34.05 | 0.82 |
|  | 2-Chloroadenine | 0.0114 ± 0.0046 | 15.32 ± 13.34 | 0.80 |
|  | N-Methyl-N-(methylsulfonyl)glycine | 0.0105 ± 0.0039 | 17.60 ± 18.74 | 0.74 |
|  | Met-Asp | 0.0096 ± 0.0031 | 17.88 ± 12.20 | 0.70 |
|  | Adrenochrome | 0.0093 ± 0.0043 | 32.88 ± 55.87 | 0.64 |
|  | N6-Acetyl-5S-hydroxy-L-lysine | 0.0088 ± 0.0036 | 21.78 ± 16.64 | 0.60 |
|  | Cyclamic acid | 0.0079 ± 0.0027 | 30.28 ± 35.65 | 0.60 |
|  | Nipecotic acid | 0.0070 ± 0.0033 | 121.12 ± 362.54 | 0.42 |
|  | 5'-Fluoro-2'-hydroxy-4-methylchalcone | 0.0067 ± 0.0034 | 48.56 ± 51.85 | 0.36 |
|  | 4-Piperidinamine, 1-(9H-purin-6-yl)- | 0.0067 ± 0.0037 | 59.32 ± 106.03 | 0.36 |
|  | 2-Hydroxyethanesulfonic acid | 0.0076 ± 0.0030 | 30.34 ± 24.71 | 0.34 |
|  | Orotidine | 0.0067 ± 0.0032 | 129.64 ± 439.56 | 0.34 |
