# Supplementary material for: Integrated machine learning for cause-of-death classification and postmortem interval prediction: Liver and kidney metabolomics from seawater-immersed rat cadavers
Source: PLoS One. 2026 Jul 23;21(7):e0353958. doi: 10.1371/journal.pone.0353958 (PMC13395348; doi:10.1371/journal.pone.0353958)
Supplement: S7 Table — Predicted PMI and prediction error are shown as mean ± SD, whereas absolute error is shown as mean value. (DOCX) [file pone.0353958.s015.docx]

**S7 Table. PMI-wise prediction bias of PMI-specific Top20 XGBoost regression models.** Predicted PMI and prediction error are shown as mean ± SD, whereas absolute error is shown as mean value.

| **Organ** | **Validation scheme** | **Actual PMI (h)** | **N** | **Predicted PMI (h), mean ± SD** | **Prediction error (h), mean ± SD** | **Absolute error (h)** |
| --- | --- | --- | --- | --- | --- | --- |
| Liver | Repeated 10-fold CV | 0 | 10 | 1.13 ± 1.27 | 1.13 ± 1.27 | 1.26 |
|  |  | 12 | 10 | 12.32 ± 3.79 | 0.32 ± 3.79 | 2.75 |
|  |  | 24 | 10 | 21.73 ± 6.64 | -2.27 ± 6.64 | 3.82 |
|  |  | 36 | 10 | 38.41 ± 5.14 | 2.41 ± 5.14 | 4.33 |
|  |  | 48 | 10 | 47.11 ± 9.75 | -0.89 ± 9.75 | 6.98 |
|  |  | 72 | 10 | 63.68 ± 6.19 | -8.32 ± 6.19 | 8.40 |
|  | Leave-one-time-point-out CV | 0 | 10 | 15.29 ± 3.18 | 15.29 ± 3.18 | 15.29 |
|  |  | 12 | 10 | 9.67 ± 9.58 | -2.33 ± 9.58 | 9.03 |
|  |  | 24 | 10 | 21.43 ± 7.68 | -2.57 ± 7.68 | 5.60 |
|  |  | 36 | 10 | 44.88 ± 7.09 | 8.88 ± 7.09 | 11.09 |
|  |  | 48 | 10 | 46.76 ± 17.49 | -1.24 ± 17.49 | 15.67 |
|  |  | 72 | 10 | 47.57 ± 0.80 | -24.43 ± 0.80 | 24.43 |
| Kidney | Repeated 10-fold CV | 0 | 10 | 0.71 ± 0.96 | 0.71 ± 0.96 | 0.86 |
|  |  | 12 | 10 | 11.18 ± 4.47 | -0.82 ± 4.47 | 3.09 |
|  |  | 24 | 10 | 22.50 ± 3.88 | -1.50 ± 3.88 | 2.40 |
|  |  | 36 | 10 | 36.59 ± 4.37 | 0.59 ± 4.37 | 3.30 |
|  |  | 48 | 10 | 48.59 ± 6.49 | 0.59 ± 6.49 | 4.09 |
|  |  | 72 | 10 | 63.01 ± 6.06 | -8.99 ± 6.06 | 9.02 |
|  | Leave-one-time-point-out CV | 0 | 10 | 11.99 ± 0.59 | 11.99 ± 0.59 | 11.99 |
|  |  | 12 | 10 | 7.99 ± 7.42 | -4.01 ± 7.42 | 7.63 |
|  |  | 24 | 10 | 20.00 ± 10.28 | -4.00 ± 10.28 | 10.52 |
|  |  | 36 | 10 | 32.31 ± 10.99 | -3.69 ± 10.99 | 10.78 |
|  |  | 48 | 10 | 50.16 ± 17.07 | 2.16 ± 17.07 | 15.39 |
|  |  | 72 | 10 | 46.47 ± 3.46 | -25.53 ± 3.46 | 25.53 |
