# Supplementary material for: Integrated machine learning for cause-of-death classification and postmortem interval prediction: Liver and kidney metabolomics from seawater-immersed rat cadavers
Source: PLoS One. 2026 Jul 23;21(7):e0353958. doi: 10.1371/journal.pone.0353958 (PMC13395348; doi:10.1371/journal.pone.0353958)
Supplement: S8 Table — PMI, postmortem interval; FDR, false discovery rate. P values and FDR-adjusted P values are reported as <0.001 for readability. (DOCX) [file pone.0353958.s016.docx]

**S8 Table. Spearman correlations between PMI and the global top-20 metabolites in liver and kidney.** PMI, postmortem interval; FDR, false discovery rate. P values and FDR-adjusted P values are reported as <0.001 for readability.

| **Organ** | **Rank** | **Metabolite** | **Spearman ρ** | **\|ρ\|** | **P value** | **FDR-adjusted P value** | **Direction vs PMI** |
| --- | --- | --- | --- | --- | --- | --- | --- |
| Liver | 1 | 10-Hydroxy-7,9-dimethyl-1,3,4,4a,7,7a-hexahydrobenzo[e]naphthalene-2,8,11-trione | 0.962 | 0.962 | <0.001 | <0.001 | Increasing |
|  | 2 | 4-(Methylamino)butanoic acid | 0.961 | 0.961 | <0.001 | <0.001 | Increasing |
|  | 3 | Formylmethionine | 0.954 | 0.954 | <0.001 | <0.001 | Increasing |
|  | 4 | N-Acetylmethionine | 0.954 | 0.954 | <0.001 | <0.001 | Increasing |
|  | 5 | Cytidine 3'-monophosphate | 0.951 | 0.951 | <0.001 | <0.001 | Increasing |
|  | 6 | 1-(4-Hydroxyphenyl)-2-methylaminoethanone | 0.950 | 0.950 | <0.001 | <0.001 | Increasing |
|  | 7 | 4,5-Dimethoxy-2,3-dihydro-1H-isoindole-1,3-dione | 0.950 | 0.950 | <0.001 | <0.001 | Increasing |
|  | 8 | 1H-Benzimidazole, 2-[[4-(5-ethyl-4-pyrimidinyl)-1-piperazinyl]methyl]-6-(trifluoromethyl)- | 0.949 | 0.949 | <0.001 | <0.001 | Increasing |
|  | 9 | N-Acetyltryptophan | 0.948 | 0.948 | <0.001 | <0.001 | Increasing |
|  | 10 | 2-{[(4-Fluorophenyl)amino]methyl}phenol | 0.948 | 0.948 | <0.001 | <0.001 | Increasing |
|  | 11 | N-Acetyl-asparagine | 0.948 | 0.948 | <0.001 | <0.001 | Increasing |
|  | 12 | Tyrosine O-sulfate | 0.947 | 0.947 | <0.001 | <0.001 | Increasing |
|  | 13 | N-Acetylaspartic acid | 0.946 | 0.946 | <0.001 | <0.001 | Increasing |
|  | 14 | Acetylleucine | 0.946 | 0.946 | <0.001 | <0.001 | Increasing |
|  | 15 | Asp-Ala | 0.943 | 0.943 | <0.001 | <0.001 | Increasing |
|  | 16 | 2-Methoxy-N-(1H-tetraazol-5-yl)benzamide | 0.943 | 0.943 | <0.001 | <0.001 | Increasing |
|  | 17 | N-Acetylphenylalanine | 0.943 | 0.943 | <0.001 | <0.001 | Increasing |
|  | 18 | 5'-Fluoro-2'-hydroxy-4-methoxychalcone | 0.941 | 0.941 | <0.001 | <0.001 | Increasing |
|  | 19 | 3,4'-Dihydroxypropiophenone | 0.940 | 0.940 | <0.001 | <0.001 | Increasing |
|  | 20 | (3,5-Dimethylphenyl)methanesulfonic acid | 0.939 | 0.939 | <0.001 | <0.001 | Increasing |
| Kidney | 1 | 6-Dimethylaminopurine | 0.965 | 0.965 | <0.001 | <0.001 | Increasing |
|  | 2 | O-Methyl-N,N'-diisopropylisourea | 0.960 | 0.960 | <0.001 | <0.001 | Increasing |
|  | 3 | Ile-Pro | 0.952 | 0.952 | <0.001 | <0.001 | Increasing |
|  | 4 | beta-Estradiol | 0.950 | 0.950 | <0.001 | <0.001 | Increasing |
|  | 5 | alpha-Estradiol | 0.950 | 0.950 | <0.001 | <0.001 | Increasing |
|  | 6 | 2-(2-Methyl-1H-imidazol-1-yl)-1-propanol | 0.947 | 0.947 | <0.001 | <0.001 | Increasing |
|  | 7 | Cotinine_N-oxide | 0.947 | 0.947 | <0.001 | <0.001 | Increasing |
|  | 8 | Hesperetin dihydrochalcone | 0.944 | 0.944 | <0.001 | <0.001 | Increasing |
|  | 9 | Biliverdin | 0.944 | 0.944 | <0.001 | <0.001 | Increasing |
|  | 10 | 13,14-Dehydro-15-cyclohexylcarbaprostacyclin | 0.944 | 0.944 | <0.001 | <0.001 | Increasing |
|  | 11 | 2-[(5,6-Diphenylfuro[2,3-d]pyrimidin-4-yl)amino]ethanol | 0.943 | 0.943 | <0.001 | <0.001 | Increasing |
|  | 12 | 2-Hydroxyhexanoic acid | 0.942 | 0.942 | <0.001 | <0.001 | Increasing |
|  | 13 | 2-Ethyl-2-hydroxybutyric acid | 0.942 | 0.942 | <0.001 | <0.001 | Increasing |
|  | 14 | Hydroxyisocaproic acid | 0.942 | 0.942 | <0.001 | <0.001 | Increasing |
|  | 15 | (3-Carboxypropyl)trimethylammonium cation | 0.942 | 0.942 | <0.001 | <0.001 | Increasing |
|  | 16 | (2S)-(+)-5,5-Dimethyl-2-morpholineacetic acid | 0.942 | 0.942 | <0.001 | <0.001 | Increasing |
|  | 17 | N-Acetylaspartylglutamate (NAAG) | 0.937 | 0.937 | <0.001 | <0.001 | Increasing |
|  | 18 | N-Acetylvaline | 0.936 | 0.936 | <0.001 | <0.001 | Increasing |
|  | 19 | 2-Methylbutyrylglycine | 0.936 | 0.936 | <0.001 | <0.001 | Increasing |
|  | 20 | 1,8-Diaminonaphthalene | 0.936 | 0.936 | <0.001 | <0.001 | Increasing |
