# Supplementary material for: Integrated machine learning for cause-of-death classification and postmortem interval prediction: Liver and kidney metabolomics from seawater-immersed rat cadavers
Source: PLoS One. 2026 Jul 23;21(7):e0353958. doi: 10.1371/journal.pone.0353958 (PMC13395348; doi:10.1371/journal.pone.0353958)
Supplement: S9 Table — The table lists the 20 most frequently selected metabolites in liver and kidney across 50 training folds. (DOCX) [file pone.0353958.s017.docx]

**S9 Table. Selection frequency of PMI-specific metabolites across repeated cross-validation folds.** The table lists the 20 most frequently selected metabolites in liver and kidney across 50 training folds.

| **Organ** | **Rank** | **Metabolite** | **Selection count** | **Selection frequency** |
| --- | --- | --- | --- | --- |
| Liver | 1 | 10-Hydroxy-7,9-dimethyl-1,3,4,4a,7,7a-hexahydrobenzo[e]naphthalene-2,8,11-trione | 50/50 | 1.00 |
|  | 2 | 4-(Methylamino)butanoic acid | 50/50 | 1.00 |
|  | 3 | N-Acetylmethionine | 50/50 | 1.00 |
|  | 4 | Formylmethionine | 50/50 | 1.00 |
|  | 5 | Cytidine 3'-monophosphate | 50/50 | 1.00 |
|  | 6 | 1-(4-Hydroxyphenyl)-2-methylaminoethanone | 50/50 | 1.00 |
|  | 7 | 4,5-Dimethoxy-2,3-dihydro-1H-isoindole-1,3-dione | 50/50 | 1.00 |
|  | 8 | 1H-Benzimidazole, 2-[[4-(5-ethyl-4-pyrimidinyl)-1-piperazinyl]methyl]-6-(trifluoromethyl)- | 50/50 | 1.00 |
|  | 9 | N-Acetyl-asparagine | 50/50 | 1.00 |
|  | 10 | Tyrosine O-sulfate | 50/50 | 1.00 |
|  | 11 | 2-{[(4-Fluorophenyl)amino]methyl}phenol | 49/50 | 0.98 |
|  | 12 | N-Acetyltryptophan | 49/50 | 0.98 |
|  | 13 | Acetylleucine | 49/50 | 0.98 |
|  | 14 | N-Acetylaspartic acid | 49/50 | 0.98 |
|  | 15 | Asp-Ala | 42/50 | 0.84 |
|  | 16 | N-Acetylphenylalanine | 42/50 | 0.84 |
|  | 17 | 2-Methoxy-N-(1H-tetraazol-5-yl)benzamide | 38/50 | 0.76 |
|  | 18 | 5'-Fluoro-2'-hydroxy-4-methoxychalcone | 29/50 | 0.58 |
|  | 19 | (3,5-Dimethylphenyl)methanesulfonic acid | 26/50 | 0.52 |
|  | 20 | 3,4'-Dihydroxypropiophenone | 23/50 | 0.46 |
| Kidney | 1 | 6-Dimethylaminopurine | 50/50 | 1.00 |
|  | 2 | O-Methyl-N,N'-diisopropylisourea | 50/50 | 1.00 |
|  | 3 | Ile-Pro | 50/50 | 1.00 |
|  | 4 | alpha-Estradiol | 50/50 | 1.00 |
|  | 5 | beta-Estradiol | 50/50 | 1.00 |
|  | 6 | 2-(2-Methyl-1H-imidazol-1-yl)-1-propanol | 50/50 | 1.00 |
|  | 7 | Cotinine_N-oxide | 50/50 | 1.00 |
|  | 8 | 13,14-Dehydro-15-cyclohexylcarbaprostacyclin | 47/50 | 0.94 |
|  | 9 | Hesperetin dihydrochalcone | 46/50 | 0.92 |
|  | 10 | Biliverdin | 45/50 | 0.90 |
|  | 11 | (3-Carboxypropyl)trimethylammonium cation | 45/50 | 0.90 |
|  | 12 | 2-Hydroxyhexanoic acid | 45/50 | 0.90 |
|  | 13 | (2S)-(+)-5,5-Dimethyl-2-morpholineacetic acid | 43/50 | 0.86 |
|  | 14 | 2-Ethyl-2-hydroxybutyric acid | 43/50 | 0.86 |
|  | 15 | 2-[(5,6-Diphenylfuro[2,3-d]pyrimidin-4-yl)amino]ethanol | 42/50 | 0.84 |
|  | 16 | Hydroxyisocaproic acid | 41/50 | 0.82 |
|  | 17 | 1,8-Diaminonaphthalene | 24/50 | 0.48 |
|  | 18 | 1,4'-Bipiperidin-3-ol | 22/50 | 0.44 |
|  | 19 | N-Acetylaspartylglutamate (NAAG) | 21/50 | 0.42 |
|  | 20 | N-Acetylvaline | 21/50 | 0.42 |
