# Supplementary material for: Integrated machine learning for cause-of-death classification and postmortem interval prediction: Liver and kidney metabolomics from seawater-immersed rat cadavers
Source: PLoS One. 2026 Jul 23;21(7):e0353958. doi: 10.1371/journal.pone.0353958 (PMC13395348; doi:10.1371/journal.pone.0353958)
Supplement: S10 Table — The table lists the 20 metabolites with the highest mean absolute TreeSHAP values in liver and kidney, together with their mean TreeSHAP ranks and top-three and top-five frequencies. (DOCX) [file pone.0353958.s018.docx]

**S10 Table. Fold-level TreeSHAP stability of PMI-specific metabolites.** The table lists the 20 metabolites with the highest mean absolute TreeSHAP values in liver and kidney, together with their mean TreeSHAP ranks and top-three and top-five frequencies.

| **Organ** | **Rank** | **Metabolite** | **Mean absolute SHAP ± SD** | **Mean SHAP rank ± SD** | **Top-3 frequency** | **Top-5 frequency** |
| --- | --- | --- | --- | --- | --- | --- |
| Liver | 1 | 10-Hydroxy-7,9-dimethyl-1,3,4,4a,7,7a-hexahydrobenzo[e]naphthalene-2,8,11-trione | 11.102 ± 4.164 | 1.24 ± 0.66 | 0.98 | 1.00 |
|  | 2 | 4-(Methylamino)butanoic acid | 2.914 ± 3.102 | 4.18 ± 2.12 | 0.46 | 0.74 |
|  | 3 | 4,5-Dimethoxy-2,3-dihydro-1H-isoindole-1,3-dione | 2.386 ± 1.437 | 3.78 ± 2.11 | 0.56 | 0.88 |
|  | 4 | 2-Methoxy-N-(1H-tetraazol-5-yl)benzamide | 2.201 ± 1.180 | 4.71 ± 3.65 | 0.44 | 0.58 |
|  | 5 | N-Acetyltyrosine | 2.069 ± 2.906 | 10.00 ± 11.31 | 0.02 | 0.02 |
|  | 6 | Cytidine 3'-monophosphate | 1.265 ± 0.780 | 6.46 ± 3.20 | 0.14 | 0.48 |
|  | 7 | 2-Amino-5-ethyl-6-methyl-4(3H)-pyrimidinone | 1.224 ± NA | 6.00 ± NA | 0.00 | 0.00 |
|  | 8 | N-Acetylmethionine | 1.051 ± 2.579 | 10.38 ± 4.22 | 0.08 | 0.14 |
|  | 9 | (3,5-Dimethylphenyl)methanesulfonic acid | 1.009 ± 0.831 | 7.54 ± 3.04 | 0.04 | 0.12 |
|  | 10 | N-Acetyl-asparagine | 0.974 ± 0.721 | 7.84 ± 3.52 | 0.04 | 0.26 |
|  | 11 | L-N-(1H-Indol-3-ylacetyl)glutamic_acid | 0.968 ± 0.684 | 8.00 ± 2.83 | 0.00 | 0.00 |
|  | 12 | 2-{[(4-Fluorophenyl)amino]methyl}phenol | 0.958 ± 0.644 | 7.20 ± 2.72 | 0.08 | 0.26 |
|  | 13 | 2-Ethyl-2-hydroxybutyric acid | 0.938 ± 0.596 | 8.14 ± 3.18 | 0.00 | 0.02 |
|  | 14 | Desaminotyrosine | 0.899 ± 1.336 | 10.33 ± 7.23 | 0.02 | 0.02 |
|  | 15 | Batimastat | 0.850 ± NA | 9.00 ± NA | 0.00 | 0.00 |
|  | 16 | N-(2-Hydroxy-3-methylbutanoyl)leucine | 0.767 ± 0.638 | 8.67 ± 4.62 | 0.00 | 0.00 |
|  | 17 | N-Acetyltryptophan | 0.703 ± 0.688 | 9.16 ± 3.81 | 0.04 | 0.20 |
|  | 18 | 3-Methoxyphenylacetic acid | 0.665 ± 0.872 | 10.33 ± 4.73 | 0.00 | 0.02 |
|  | 19 | 5'-Fluoro-2'-hydroxy-4-methoxychalcone | 0.648 ± 0.407 | 8.62 ± 2.68 | 0.02 | 0.04 |
|  | 20 | Leu-Leu | 0.626 ± 0.671 | 9.00 ± 4.24 | 0.00 | 0.00 |
| Kidney | 1 | Ile-Pro | 6.315 ± 4.700 | 3.12 ± 3.56 | 0.70 | 0.88 |
|  | 2 | Biliverdin | 3.711 ± 1.720 | 2.89 ± 1.45 | 0.66 | 0.82 |
|  | 3 | beta-Estradiol | 3.039 ± 2.707 | 4.92 ± 3.62 | 0.34 | 0.72 |
|  | 4 | Hesperetin dihydrochalcone | 2.902 ± 4.399 | 8.52 ± 5.63 | 0.28 | 0.34 |
|  | 5 | O-Methyl-N,N'-diisopropylisourea | 2.613 ± 1.268 | 4.06 ± 2.08 | 0.58 | 0.76 |
|  | 6 | Gabapentin | 2.152 ± 1.348 | 5.00 ± 2.71 | 0.02 | 0.06 |
|  | 7 | 6-Dimethylaminopurine | 1.936 ± 1.357 | 5.54 ± 2.57 | 0.24 | 0.56 |
|  | 8 | 2-Hydroxyhexanoic acid | 1.729 ± 4.490 | 12.31 ± 5.26 | 0.10 | 0.14 |
|  | 9 | Harman | 1.144 ± 0.653 | 6.62 ± 2.13 | 0.00 | 0.06 |
|  | 10 | 1-(2-Methoxyethyl)piperazine | 0.945 ± 2.219 | 12.33 ± 5.45 | 0.02 | 0.02 |
|  | 11 | 1,8-Diaminonaphthalene | 0.926 ± 0.909 | 8.46 ± 2.47 | 0.00 | 0.06 |
|  | 12 | 2-[(5,6-Diphenylfuro[2,3-d]pyrimidin-4-yl)amino]ethanol | 0.838 ± 0.760 | 8.79 ± 3.30 | 0.02 | 0.12 |
|  | 13 | alpha-Estradiol | 0.777 ± 0.931 | 11.58 ± 5.76 | 0.02 | 0.16 |
|  | 14 | Tyr-Pro | 0.760 ± 0.424 | 9.50 ± 1.73 | 0.00 | 0.00 |
|  | 15 | 2-Pyridinemethanamine, N-[[4-(1,4,8,11-tetraazacyclotetradec-1-ylmethyl)phenyl]methyl]- | 0.712 ± 0.973 | 12.00 ± 5.16 | 0.00 | 0.00 |
|  | 16 | 2-Methylbutylamine | 0.703 ± NA | 9.00 ± NA | 0.00 | 0.00 |
|  | 17 | 1,4'-Bipiperidin-3-ol | 0.620 ± 0.715 | 11.27 ± 4.32 | 0.00 | 0.04 |
|  | 18 | 1-Methyl-5-imidazoleacetic_acid | 0.600 ± 0.754 | 10.33 ± 4.18 | 0.00 | 0.02 |
|  | 19 | 1-Ethyl-5-methyl[1,2,4]triazolo[4,3-a]quinolin-8-yl methyl ether | 0.596 ± NA | 6.00 ± NA | 0.00 | 0.00 |
|  | 20 | 3-Aminoquinoline | 0.565 ± 1.079 | 13.33 ± 5.89 | 0.00 | 0.02 |
