# Supplementary material for: Integrated machine learning for cause-of-death classification and postmortem interval prediction: Liver and kidney metabolomics from seawater-immersed rat cadavers
Source: PLoS One. 2026 Jul 23;21(7):e0353958. doi: 10.1371/journal.pone.0353958 (PMC13395348; doi:10.1371/journal.pone.0353958)
Supplement: S11 Table — (DOCX) [file pone.0353958.s019.docx]

**S11 Table. Distribution of metabolite annotation confidence levels in the liver and kidney datasets.**

| **Organ** | **Level 1, n (%)** | **Level 2, n (%)** | **Level 3, n (%)** | **Level 3.1, n (%)** | **Level 3.2, n (%)** | **Total, n** |
| --- | --- | --- | --- | --- | --- | --- |
| Liver | 529 (20.9) | 1,845 (73.0) | 153 (6.1) | 97 (3.8) | 56 (2.2) | 2,527 |
| Kidney | 559 (19.3) | 2,155 (74.3) | 188 (6.5) | 109 (3.8) | 79 (2.7) | 2,902 |
